# A peptide mimic of SOCS1 modulates equine peripheral immune cells *in vitro* and ocular effector functions *in vivo*: implications for recurrent uveitis

**DOI:** 10.3389/fimmu.2024.1513157

**Published:** 2025-01-10

**Authors:** Lauren Stewart Stafford, Caryn E. Plummer, W. Clay Smith, Daniel J. Gibson, Jatin Sharma, Valeria Vicuna, Sisse Diakite, Joseph Larkin

**Affiliations:** ^1^ Microbiology and Cell Science, Institute of Food and Agricultural Science, University of Florida, Gainesville, FL, United States; ^2^ Departments of Large and Small Animal Clinical Sciences, College of Veterinary Medicine, University of Florida, Gainesville, FL, United States; ^3^ Department of Ophthalmology, College of Medicine, University of Florida, Gainesville, FL, United States; ^4^ Capstone College of Nursing, University of Alabama, Tuscaloosa, AL, United States

**Keywords:** suppressor of cytokine signaling 1 (SOCS1), recurrent uveitis, equine (horse), spontaneous model, ocular immunity, PBMC (peripheral blood mononucleated cells), tumor necrosing factor alpha, interleukins

## Abstract

**Introduction:**

Recurrent uveitis (RU), an autoimmune disease, is a leading cause of ocular detriment in humans and horses. Equine and human RU share many similarities including spontaneous disease and aberrant cytokine signaling. Reduced levels of SOCS1, a critical regulator of cytokine signaling, is associated with several autoimmune diseases. Topical administration of SOCS1-KIR, a peptide mimic of SOCS1, was previously correlated to reduced ocular pathologies within ERU patients.

**Methods:**

To further assess the translational potential of a SOCS1 mimetic to treat RU, we assessed peptide-mediated modulation of immune functions *in vitro*, using equine peripheral blood mononuclear cells (PBMC), and *in vivo* through topical administration of SOCS1-KIR into the eyes of experimental (non-uveitic) horses. Equine PBMCs from non-uveitic control and ERU horses were cultured with or without SOCS1-KIR pretreatment, followed by 72 hours of mitogen stimulation. Proliferation was assessed using MTT, and cytokine production within cell supernatants was assessed by Luminex. SOCS1-KIR or carrier eye-drops were topically applied to experimental horse eyes twice daily for 21 days, followed by enucleation and isolation of ocular aqueous and vitreous humor. Histology was used to assess peptide treatment safety and localization within treated equine eyes. Cytokine secretion within aqueous humor and vitreous, isolated from experimental equine eyes, was measured by Luminex.

**Results:**

Following SOCS1-KIR pretreatment, cell proliferation significantly decreased in control, but not ERU-derived PBMCs. Despite differential regulation of cellular proliferation, SOCS1-KIR significantly reduced TNFα and IL-10 secretion in PHA-stimulated control and ERU equine PBMC. SOCS1-KIR increased PBMC secretion of IL-8. Topically administered SOCS1-KIR was well tolerated. Although SOCS1-KIR was undetectable within the eye, topically treated equine eyes had significant reductions in TNFα and IL-10. Interestingly, we found that while SOCS1-KIR treatment reduced TNFα and IL-10 production in healthy and ERU PBMC, SOCS1-KIR differentially modulated proliferation, IP-10 production, and RANTES within these two groups suggesting possible differences in cell types or activation status.

**Discussion:**

Topical administration of a SOCS1 peptide mimic is safe to the equine eye and reduces ERU associated cytokines IL-10 and TNFα serving as potential biomarkers of drug efficacy in a future clinical trial.

## Introduction

Equine recurrent uveitis (ERU) is the leading cause of blindness in horses (1–5). The prevalence of disease in European equine populations ranges from 5% to 15%, while in the United States, disease prevalence may approach 25% (1–6). Uveitis is defined as inflammation of the uvea, which includes the choroid, ciliary body, and iris. However, given the uvea’s intimate association with ocular structures, such as the retina, lens, drainage apparatus, and cornea, and its critical role as the predominant vascular supplier to the interior of the eye, inflammation of the uvea commonly results in damage to these structures concurrently (7, 8). ERU is characterized by either remitting/relapsing or chronic insidious intraocular inflammation, which ultimately results in ocular damage and sight deficits. Due to the recurrent and relapsing nature of the disease, attacks accumulate in the eye, resulting in severe ocular detriment and even full vision loss (3). Unfortunately, due to financial, safety, utility, and ethical concerns, affected horses are often euthanized (2, 9). One study found that the median time from enrollment to euthanasia (because of ERU) was 3.5 years (10).

Recurrent uveitis (RU) is also a leading cause of blindness in humans worldwide, accounting for nearly 10% of blindness and visual handicap cases in the United States (2, 11, 12). RU is a devastating ocular disease characterized by relapsing bouts of intraocular inflammation. Due to similarities in immunological and clinical features, ERU acts as the only spontaneous model of recurrent, non-infectious uveitis in humans (2, 3, 13–17). While rodent models of induced uveitis can be used to gain an understanding of the mechanisms of disease, spontaneous ERU more accurately recapitulates human disease. There are several advantages of using an equine model, including the similar kinetics of human disease, the long lifespan of horses, the large globe size of the horse compared to that of traditional small laboratory models, and the volume of equine ocular material (3, 14, 18).

Typical clinical presentations of RU include pain, vascular congestion and hyperemia, corneal edema, aqueous flare, hypopyon (leukocytic exudate), hyphema, miosis, and lowered intraocular pressure (1, 8, 13, 14, 19–22). Infiltrating T cells mediate the breakdown of the blood–ocular barrier and drive the chronic inflammation of the eye. Additional sight-threatening conditions associated with RU include synechia, retinal destruction and detachment, and corneal scarring (1, 8, 14, 20). Often secondary to chronic uveitis, conditions like glaucoma, cataracts, and phthisis bulbi can lead to permanent vision loss and blindness in humans and horses (1, 8, 13, 19, 20, 23–25). When treating ERU, the therapeutic goals are to reduce damage-inducing inflammation, alleviate pain and discomfort, and preserve vision (3, 22, 26). The most common treatment applications for RU include the use of immunosuppressive drugs and, to a lesser extent, surgeries such as vitrectomy. Immunosuppressants, like corticosteroids and calcineurin inhibitors (such as cyclosporine), work to reduce the inflammatory response in the eye but unfortunately do not eliminate either the inflammatory process or the stimulus thereof. Additionally, corticosteroids have several adverse effects with long-term use. These include the development of cataracts, glaucoma, and immunosuppression, which increases the risk of infection (2, 7, 26). Vitrectomy, which surgically removes debris and inflammatory cells from the vitreous humor (decreasing the burden of inflammatory signaling), has limited efficacy and requires advanced training and surgical expertise to be performed (3). Many patients also become refractory to standard-of-care strategies due to the chronic and persistent nature of the disease. As such, there remains a critical need to develop novel, efficacious, and safe drugs to prevent or significantly delay ocular decrement related to RU.

While the etiology of RU is not well understood, as previously noted, pathogenesis appears to be driven by activated CD4^+^ T helper cells (specifically Th1 and Th17 cells) that inappropriately track to the eye (1, 2, 27, 28). Th1 and Th17 cells secrete proinflammatory cytokines that can cause localized tissue damage, as well as activate other cell types. While Th1 cells drive inflammatory processes in macrophages, through the secretion of TNFα and IFNγ (12, 13), the production of IL-6 and IL-17 by Th17 cells can enhance inflammation through chemokine secretion (29, 30). Additionally, aberrant TNFα signaling can promote the destruction of ocular structures through programmed cell death and necrosis (31). As such, lymphocyte activation, modulation of cytokine levels, and regulation of cellular responsiveness to cytokines could all serve as potential targets for the treatment of RU. *In vitro* T-cell activation can be assessed using phytohemagglutinin (PHA), a mitogen that activates T lymphocytes through T-cell receptor crosslinking (32, 33).

Many cytokines utilize the Janus kinase and signal transducers and activators of transcription (JAK/STAT) pathway for cellular communication and propagation of immune processes such as inflammation (34–36). Indeed, the JAK/STAT pathway is necessary for T lymphocyte signaling, maintenance, activation, and differentiation (7). Naïve CD4^+^ T helper cells differentiate into Th1 and Th17 cells by actions of STAT1 and STAT3, respectively (7). Several inflammatory and autoimmune disorders are directly related to the dysregulation of the JAK/STAT pathway and its associated cytokine-inducible gene transcription. In addition to irritable bowel disease, psoriasis, and rheumatoid arthritis, JAK/STAT signal dysregulation has been implicated in RU (7, 34, 37–40). As such, targeting this pathway could lead to positive outcomes in patients.

Suppressors of cytokine signaling (SOCS) are a family of intracellular proteins that act as negative regulators of the JAK/STAT signaling cascade (41, 42). SOCS proteins moderate cytokine signaling by limiting the intensity and duration of subsequent cellular and metabolic effector functions. SOCS proteins act through the SOCS box, which targets critical intracellular signaling components for proteasomal degradation through ubiquitination. In addition to the SOCS box, SOCS1 and SOCS3 also contain a kinase inhibitory region (KIR) that can inhibit the activation of STATs by directly binding to Janus kinases (35, 43). The KIR of SOCS1, which can inhibit kinase activity in the absence of the SOCS box (7), and its respective binding groove on JAK2 are highly conserved across several relevant mammalian species, including humans, mice, and horses (2). Significantly, we and others have demonstrated that peptide mimics of the KIR region of SOCS1 can inhibit immune activation and disease progression in *in vitro* and murine models of disease (44–47), including induced models of uveitis. In experimental mouse models of uveitis, topical administration of the peptide resulted in retinal protection (7, 11), modulation of T lymphocyte function (7), and alterations in cytokine production (43, 48, 49). However, evidence demonstrating the potential to translate these important findings from the bench to the bedside is limited. Significantly, we have shown that the topical administration of a SOCS1-KIR mimetic peptide to the eye of ERU horses significantly reduced ERU-associated discomfort, hyperemia, and aqueous flare (2).

Although promising, questions associated with mechanisms of action and a safe, efficacious dose range in the equine eye remain. In this study, we aimed to investigate the immunomodulatory effects of SOCS1-KIR administration on equine peripheral blood mononuclear cells (PBMCs) under mitogen stimulation through cell proliferation and cytokine secretion assays. We also investigated the safety of topical SOCS1-KIR administrations to the equine eye using healthy horses and assayed cytokine regulation by the peptide. We found significant reductions in IL-10 and TNFα secretion in SOCS1-KIR-treated PBMC cells *in vitro*, under PHA stimulation, and *in vivo* in the aqueous humor of SOCS1-KIR-treated equine eyes. We believe this is the first report of the immunomodulatory effects of SOCS1-KIR mimetic in the equine eye and suggest that IL-10 and TNFα may serve as potential biomarkers for SOCS1-KIR immunomodulation.

## Materials and methods

### Evaluation of topical SOCS1-KIR for safety in equine eye

Nine healthy grade (mixed breed) horses (lacking ERU) were recruited to assess the safety of the peptide in the equine eye. *A priori* power analysis established that four equine subjects (a total of eight eyes) would be sufficient to assess a significant change in electroretinography (ERG) measurements, based on historical data from the institution. No exclusion criteria were made in obtaining healthy, experimental horses beyond the absence of active, or historical evidence, of inflammatory ocular disease. An investigator (C.P.) was masked during administration, receiving deidentified peptide and placebo control groups. Horses initially received complete ophthalmic and physical examinations and received ERG in both eyes to establish baseline profiles. Three horses received 0.2 mg of either SOCS1-KIR or vehicle in either the right or left eye, three horses received 1 mg, and three horses received 2 mg twice daily for 21 days to determine the maximum effective dose of topical administration. Doses were administered in 100-μL total volume. Comparisons between eyes, either receiving peptide treatment or not, were made both in each horse and between horses to assess safety. ERGs, measuring α- and β-wave potentials, and flicker responses were performed on days 0, 1, 7, 14, and 21. Complete physical and ophthalmic examinations were also performed on days 0, 1, 7, 14, and 21 to assess SOCS1 KIR-mediated changes to ocular structure and function. Safety outcomes at each observation period included the following: i) ocular irritation scores (utilizing the modified McDonald–Shadduck scoring system), ii) number of new cases of ocular infections, iii) number of horses affected with peptide-mediated damage to the eye, iv) intraocular pressure (mmHg), and v) ERG b-wave amplitudes to assess retinal function. At 21 days, after ERG and examinations, experimental horses were euthanized humanely by intravenous pentobarbital, followed by histopathological examination of the SOCS1-KIR- or carrier-treated eyes. In addition to isolating and submitting portions of the enucleated eyes to core facilities for independent evaluation, aqueous and vitreous humor were collected and frozen for future use.

### Equine peripheral blood collection and PBMC separation

Whole blood from 17 healthy (non-ERU) horses and 10 ERU-affected horses were collected in heparin-coated vacutainer collection tubes for peripheral blood mononuclear cell isolation.

PBMCs were separated using the Ficoll-Paque Plus (GE Healthcare, Chicago, IL, USA) protocol. Equine whole blood with a volume of 15–20 mL was poured into a 50-mL conical tube. Equal parts of 1× Dulbecco’s phosphate-buffered saline (DPBS) were added to the blood. Using two 9-in. glass Pasteur pipettes placed into the bottom of the conical tube, Ficoll was added to reach 50-mL total volume and centrifuged at 1,200 × *g* for 20 minutes. After aspirating the plasma layer, the interphase (buffy coat) layer was removed and transferred to a new 50-mL conical tube. To reach 50-mL total volume, 1× DPBS was then used and centrifuged at 450 × *g* for 10 minutes. The supernatant was fully aspirated, and the pellet was resuspended in 1 mL 1× DPBS. The resuspended pellet was then used to acquire the cell count.

### IACUC statement

All procedures performed on animals were approved by the Institutional Animal Care and Use Committee (IACUC) of the University of Florida and were conducted in strict accordance with the approved guidelines.

### PBMC cell culture

In 96-well plates, 100 μL of equine PBMCs at a cell concentration of 1–4 × 10^6^ cells/mL in PBMC media containing RPMI 1640 (Corning, New York, NY, USA) supplemented with 10% fetal bovine serum (FBS) and 1× penicillin–streptomycin–neomycin (PSN) was plated. The PBMCs were subsequently pre-incubated in the presence or absence of 33 μM SOCS1-KIR mimetic peptide (DTHFRTFRSHSDYRRIGGGGGDTHFRTFRSHSDYRRI) for 2 hours, followed by stimulation with graded doses of lipopolysaccharide (LPS) (0 μg/mL, 1 μg/mL, 10 μg/mL, and 100 μg/mL) or PHA (0 μg/mL, 1 μg/mL, 10 μg/mL, and 100 μg/mL) diluted in 100 μL PBMC media and incubated for 72 hours at 37°C in 5% CO_2_. After 72 hours, 100 μL of supernatant was obtained and stored for future assays.

### MTT

Cell proliferation was measured using the EMD Millipore MTT assay (EMD Millipore, Burlington, MA, USA; Catalog #CT01) utilizing the manufacturer’s instructions (50). Briefly, PBMCs (1–4 × 10^6^ cells) isolated from ERU horses or healthy controls were stimulated for 72 hours under varied conditions after which 0.01 mL AB Solution was added to each well and incubated at 37°C for 4 hours. At the end of the incubation, plates were centrifuged at 300 × *g* for 5 minutes. A 70-μL aliquot of the supernatant was obtained from each well, and 100 μL dimethyl sulfoxide (DMSO) was added to each well. Plates were incubated for 15 minutes at 37°C to dissolve crystals. After incubation, plates were read at 560 nm on an ELISA plate reader (Gen5).

### Cytokine secretion analysis

After incubation with PHA (1 μg/mL, 10 μg/mL, and 100 μg/mL) or LPS (1 μg/mL, 10 μg/mL, and 100 μg/mL), supernatants were assayed for equine IL-6 (Bio-Techne R&D SYSTEMS, Minneapolis, MN, USA; Equine IL-6 DuoSet ELISA, Catalog #DY1886) and equine TNFα (Bio-Techne R&D SYSTEMS; Equine TNF-alpha DuoSet ELISA, Catalog #DY1814) by ELISA or using an equine-specific cytokine multiplex assay (MilliporeSigma; Milliplex Equine Cytokine/Chemokine Magnetic Bead Panel-Immunology Multiplex Assay, Catalog #EQCYTMAG-93K) as per the manufacturer’s instruction. The multiplex assay measured the following equine-specific cytokine/chemokines: eotaxin, IL-1α, IL-6, IL-8, IL-10, IL-17, IFNγ, IP-10, MCP-1, RANTES, and TNFα. The Luminex data were normalized to a standard curve and analyzed using the 5P analysis method as described in Duran et al. (51).

### Immunofluorescence

SOCS1-KIR internalization was measured in HeLa cells and equine PBMCs using cytological immunofluorescence. Briefly, adherent HeLa cells were seeded at a density of 400,000 cells/mL on culture-treated chamber slides and incubated for 24 hours. Cells were then treated over a 24-hour period with SOCS1-KIR peptide (33 μM) for the following time points: 0.25 hours, 0.5 hours, 1 hour, 3 hours, 6 hours, 12 hours, and 24 hours. In the case of equine PBMCS, a suspension of equine PBMCs (1 × 10^6^ cells/mL) was gently seeded on coverslips that were then placed in small Petri dishes. Equine PBMCs were then incubated with SOCS1-KIR mimetic peptide for 24 hours. After treatment, cells were washed, fixed in 4% paraformaldehyde (in PBS), and subsequently permeabilized with PBS (Mg^2+^ and Ca^2+^) + 1% Triton + 50 mM glycine. Following permeabilization, cells were blocked in PBS (Mg^2+^ and Ca^2+^) + 50 mM glycine + 5% FBS + 0.1% TX-100. Cells were then incubated with anti-KIR rabbit sera (1:200 dilution prepared in blocking solution), washed, and then incubated in secondary antibody solution (1:250 dilution of goat anti-rabbit IgG conjugated to Alexa Fluor 488). After secondary antibody incubation, cells were washed. 4′,6-Diamidino-2-phenylindole (DAPI) and coverslips were then added to the slides. HeLa cell images were obtained, and the arithmetic mean intensity of AF488 was measured using the Zeiss ZEN microscopy software, version 3.5. For equine PBMCs, coverslips were mounted to the slides with the culture side down using DAPI mounting media. Equine PBMC images were obtained using the Leica SP5 confocal microscope. AF488 fluorescence intensity was measured using Fiji (52).

### Immunohistochemistry/histology

SOCS1-KIR- or carrier-treated equine eyes were enucleated by a veterinary professional following euthanasia. Eye tissues were then processed by removing excess tissue and placed into histology cassettes for paraffin embedding and sectioning. Once sectioned, slides were deparaffinized using Histo-Clear, followed by a dilution series of EtOH (100%–70%). Equine eye slides were processed as described in Xu et al. (53). Briefly, slides were washed in PBS for 10 minutes, followed by washes in PBS + 50 mM glycine + 0.25% TX-100 for 10 minutes, and a final PBS wash. Slides were then blocked in PBS + 2% low Ig serum + 0.1% TX-100 for 1 hour and incubated overnight with primary antibody (1:400 anti-SOCS1-KIR) in a blocking solution. The next day, slides were washed with PBS + 0.1% TX-100 three times for 5 minutes and then incubated in secondary antibody dilution 1:250 goat anti-rabbit IgG Alexa Fluor 488 (Thermo Fisher, Waltham, MA, USA; A-11008) or 1:250 donkey anti-rabbit IgG Alexa Fluor 488 (Jackson ImmunoResearch, West Grove, PA, USA; 711-545-152) and DAPI (10 μg/mL) for 2 hours. Slides were then washed with PBS + 0.1% TX-100 three times for 5 minutes. Once dry, slides and coverslips were sealed with Mowiol 4-88 mounting media (Sigma). Eye slides were imaged using a Leica SP5 confocal microscope.

### Statistical analyses

Statistical analyses and figure creation were performed using GraphPad Prism 9 (GraphPad Software). Paired t-tests were used to compare cell proliferation, cytokine secretions, and AF488 intensity in the presence or absence of SOCS1-KIR mimetic pretreatment. A representative figure is provided, which denotes TNFα secretion in the presence or absence of SOCS1-KIR for the PBMCs from each horse and t-test analysis (**Supplementary Figure 1**). Spearman’s correlations were used to analyze the relationship between cytokines and cell proliferation of control and ERU PBMCs under mitogen stimulation. Ordinary one-way ANOVA was used to study mitogen stimulation effects in equine PBMCs, increase in AF488 detection over 24 hours in HeLa cells, and dose-dependent response in aqueous humor cytokines. Fold changes were calculated using the difference in medians for each cytokine [(cytokine of interest − unstimulated))/unstimulated]. Bars are shown as mean plus standard deviation. An alpha threshold of <5% (or p < 0.05) was used for declaring statistical significance. Statistical test results are reported as p-values.

## Results

### Horse demographics

To assess the effect of SOCS1-KIR administration *in vitro*, we collected whole blood and isolated PBMCs from 27 horses. Seventeen (63%) horses acted as non-ERU controls, and 10 (37%) horses were affected with ERU. The age range for this cohort was 7 to 39 years with an average age of 23.5 years, an average age of 25.8 years in ERU, and 22.4 years in non-ERU controls. Sixteen of the horses were mares, and 11 were geldings. Thoroughbreds were the most abundant breed overall (11), but only one Thoroughbred had ERU. All Appaloosas (3/3) had ERU, and 50% of Quarter Horses had ERU (2/4) (**Table 1**).

**Table 1:** Demographics for study horse PBMC. Total N = 27 (17 healthy, 10 ERU). Values are shown as N (%) or mean +/- SD.

Horse demographics (N = 27)		N (%) or mean ± SD
Age (years)		23.5 ± 8.4
ERU		25.8 ± 12.1
Non-ERU controls		22.4 ± 6
Sex		
Mare		16 (59)
Gelding		11 (41)
Disease status		
ERU		10 (37)
Non-ERU controls		17 (63)
Breed		
Appaloosa		3 (11.1)
Quarter Horse		4 (14.8)
Thoroughbred		11 (40.7)
Standardbred		1 (3.7)
Belgian		1 (3.7)
Paso		1 (3.7)
Percheron		1 (3.7)
Bay		1 (3.7)
Palomino Pony		1 (3.7)
Mini Horse		1 (3.7)
Hanoverian		1 (3.7)
Dutch Warmblood		1 (3.7)
	Breed ERU (N = 10)	
	Appaloosa	3 (30)
	Quarter Horse	2 (20)
	Thoroughbred	1 (10)
	Standardbred	0 (0)
	Belgian	0 (0)
	Paso	0 (0)
	Percheron	0 (0)
	Bay	1 (10)
	Palomino Pony	1 (10)
	Mini Horse	1 (10)
	Hanoverian	0 (0)
	Dutch Warmblood	1 (10)

ERU, equine recurrent uveitis.

### PHA and LPS stimulation significantly affect cell proliferation and cytokine secretion

To establish the effects of mitogen stimulation on equine PBMCs, first, cells were treated with 1 μg/mL, 10 μg/mL, and 100 μg/mL of PHA or LPS for 72 hours. 3-(4,5-Dimethylthiazol-2-yl)-2,5-diphenyl-2*H*-tetrazolium bromide (MTT) was then utilized to measure cell proliferation and viability, while Luminex was used to quantify supernatant protein concentrations secreted by the equine PBMCs. MTT was reduced to purple formazan crystals by an NADPH-dependent oxidoreductase, presumably in metabolically active cells. This assay was readily used to measure changes in cell proliferation and viability (54, 55). Treatment with 1 μg/mL PHA stimulation significantly increased cell proliferation in both control (p < 0.0001, 58% increase) and ERU PBMCs (p = 0.0003; 109% increase) (**Supplementary Figure S2**). LPS, however, did not significantly affect cell proliferation in either group. TNFα, IL-10, IL-6, IFNγ, IL-8, and RANTES were all significantly increased under 1 μg/mL PHA stimulation in both control and ERU PBMCs (**Supplementary Figures 3**, **4**). Under LPS stimulation, only IL-10 and IL-8 were significantly increased in both groups (**Supplementary Figures 3**, **4**).

### SOCS1-KIR mimetic peptide administration significantly lowers cell proliferation in control but not ERU PBMCs

**Figure 1:**
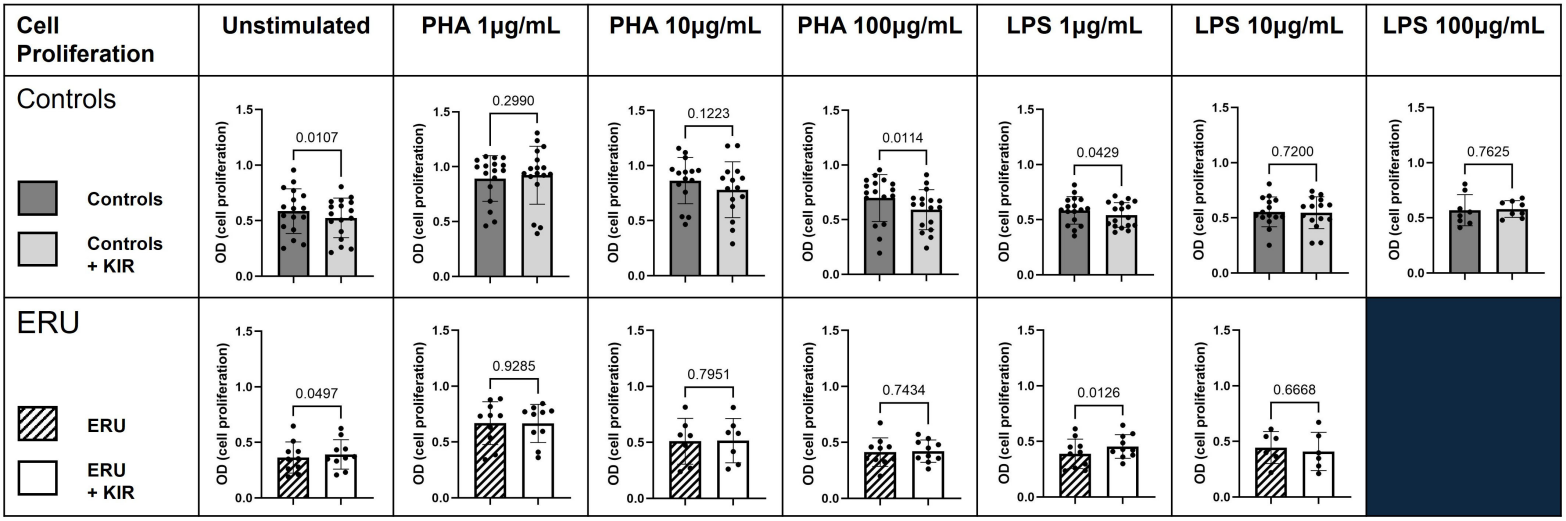
SOCS1-KIR mimetic peptide administration significantly lowers cell proliferation of non-uveitis control equine PBMCs. PBMCs isolated from either ERU horses or non-uveitis control horses were stimulated *in vitro* with either PHA (1 µg/mL, 10 µg/mL, or 100 µg/mL) or LPS (1 µg/mL, 10 µg/mL, or 100 µg/mL) in the presence or absence of SOCS1-KIR for 72 hours followed by assessment of proliferation by MTT. Shown are graphical representations where each dot represents an individual horse. Dark gray bars, control PBMCs; light gray bars, control PBMCs + SOCS1-KIR; slashed line bars, ERU PBMCs; white bars, ERU PBMCs + SOCS1-KIR. Statistical analysis = paired t-test; p-value listed above bars. Controls N = 17 horse samples; ERU N = 10 horse samples. PBMCs, peripheral blood mononuclear cells; ERU, equine recurrent uveitis; PHA, phytohemagglutinin; LPS, lipopolysaccharide; MTT, 3-(4,5-dimethylthiazol-2-yl)-2,5-diphenyl-2*H*-tetrazolium bromide.

We have previously shown that SOCS1-KIR administration modulated lymphocyte and innate immune cell activation, which was correlated to reductions in autoimmune pathology in rodent disease models (7, 11, 46, 56). As a next step in evaluating the potential of the SOCS1-KIR mimetic peptide to serve as an equine immunomodulatory agent, we assessed the ability of the peptide to modulate equine PBMC effector functions at steady state and after activation. Using the mitogens PHA (T lymphocyte stimulant) or LPS (APC activator), we first assessed the ability of SOCS1-KIR to modulate cellular proliferation of PBMCs obtained from controls or ERU horses, as measured by MTT. SOCS1-KIR significantly inhibited the proliferation of control equine PBMCs *in vitro*, as assessed by MTT, in the absence of stimulation (p-value = 0.01) and when stimulated with either PHA (100 μg/mL) (p-value = 0.01) or LPS (1 μg/mL) (p-value = 0.04). In stark contrast, SOCS1-KIR administration failed to inhibit the proliferation of PBMCs isolated from ERU horses. In fact, SOCS1-KIR administration significantly enhanced the proliferation of unstimulated or LPS-treated PBMCs from ERU horses (p-value < 0.05 in unstimulated and 1 μg/mL LPS-stimulated cells) (**Figure 1**). SOCS1-KIR administration did not significantly affect cell proliferation in 10 μg/mL PHA-, 10 μg/mL LPS-, or 100 μg/mL LPS-stimulated equine PBMCs.

### Administration of SOCS1-KIR mimetic peptide significantly lowers TNFα and IL-10 secretion under PHA stimulation

**Figure 2:**
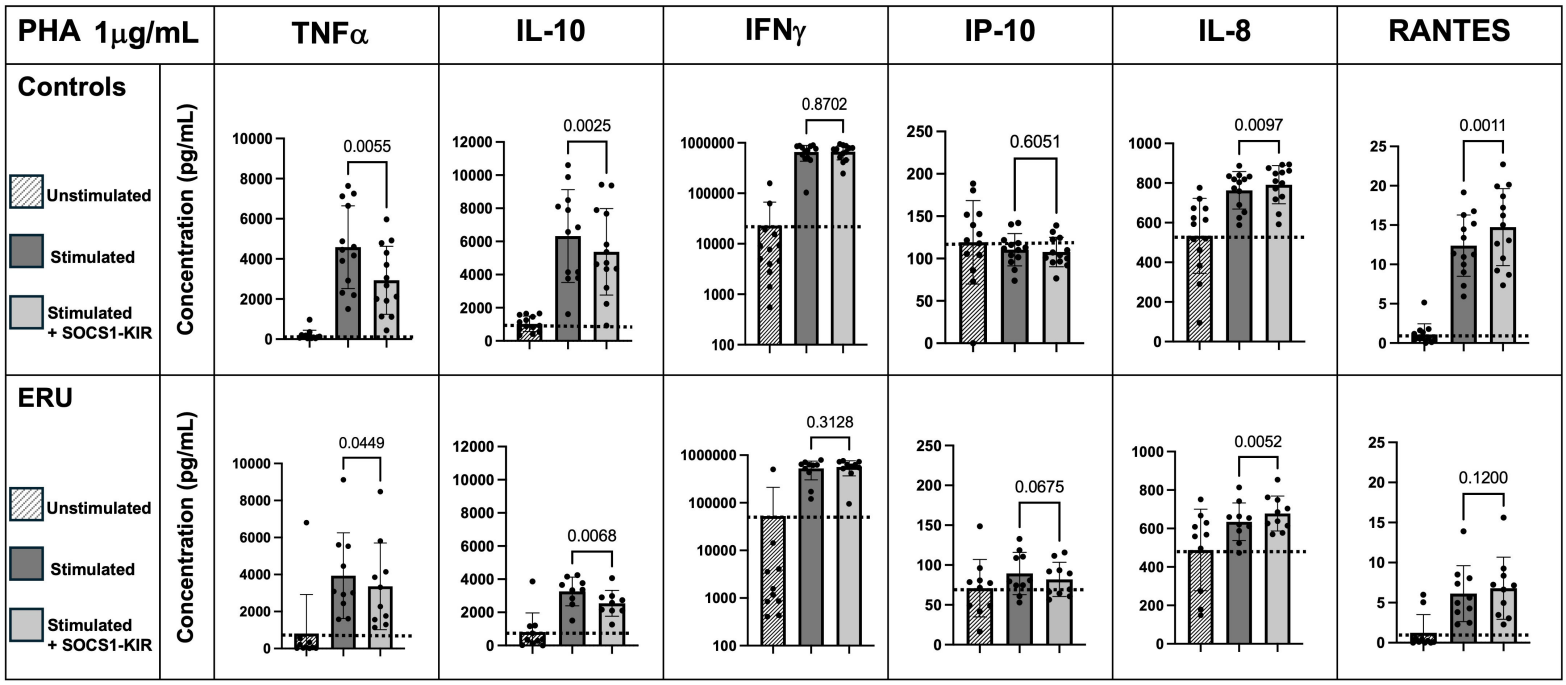
SOCS1-KIR mimetic peptide administration significantly lowers TNFα and IL-10 secretion under 1 µg/mL PHA stimulation in non-uveitic control and ERU PBMCs, while SOCS1-KIR administration increases IL-8 secretion. Slashed bars, unstimulated PBMCs; dark gray bars, in absence of SOCS1-KIR pretreatment; light gray bars, in presence of SOCS1-KIR pretreatment. Dotted line indicates increase or decrease from unstimulated baseline. Statistical analysis = paired t-test to measure effect of SOCS1-KIR pretreatment; p-value listed above bars. Controls N = 13 horse samples; ERU N = 10 horse samples. PHA, phytohemagglutinin; ERU, equine recurrent uveitis; PBMCs, peripheral blood mononuclear cells.

Given the critical role of inflammatory cytokines, such as TNFα, in the progression of both ERU (13, 57, 58) and human recurrent uveitis (59–61), we next evaluated the ability of the SOCS1-KIR mimetic to regulate cytokine production in activated equine PBMCs (untreated controls were provided here for baseline reference). Due to the limited effect of SOCS1-KIR administration on higher concentrations of mitogen stimulation, we focused on 1 μg/mL PHA or LPS. After SOCS1-KIR pretreatment, we observed a significant reduction in both non-uveitic controls and ERU PBMCs in TNFα secretion under 1 μg/mL PHA stimulation [p-value = 0.0055 (40% reduction) and 0.0449 (8% reduction), respectively] (**Figure 2**). We also observed a significant reduction in IL-10 secretion, which is a critical regulator of TNFα (62) under PHA stimulation in both controls and ERU PBMCs [p-value < 0.01 (32% and 24% reduction, respectively)] (**Figure 2**). In PHA-stimulated ERU PBMCs only, TNFα and IL-10 were significantly positively correlated, with SOCS1-KIR pretreated cells exhibiting lower concentrations of TNFα and IL-10 (p-value = 0.0003, R = 0.72) (**Supplementary Figure 5**). However, this relationship was not found in control PBMCs. While IFNγ secretion did not appear to be affected by SOCS1-KIR administration, interferon gamma-inducible protein 10 (IP-10, also known as CXCL10) was reduced in ERU samples only, although not reaching statistical significance (p < 0.068). IFNγ and IP-10 were also significantly correlated under PHA stimulation in both ERU and control PBMCs (p-values = 0.00069 and 0.00286, R = 0.84 and 0.85, respectively) (**Supplementary Tables 1**, **2**). Notably, RANTES (also known as CCL5) secretion was significantly increased in controls upon the addition of SOCS1-KIR under PHA stimulation [p = 0.001 (32% increase)].

### SOCS1-KIR administration significantly lowers IFNγ, RANTES, and IP-10 secretion in LPS-stimulated PBMCs from ERU horses

**Figure 3:**
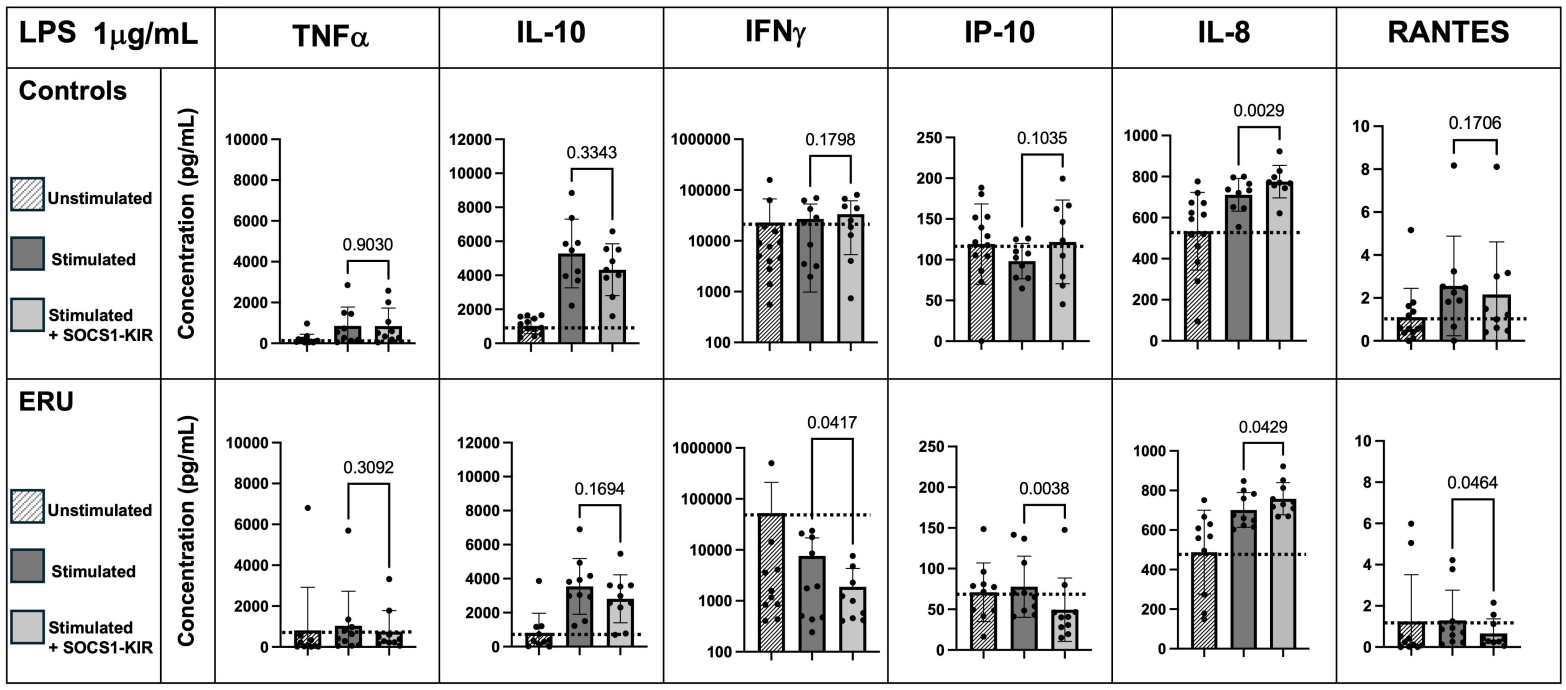
SOCS1-KIR mimetic peptide administration significantly lowers IFNγ, IP-10, and RANTES secretion under 1 µg/mL LPS stimulation in ERU PBMCs, while IL-8 is significantly increased. Slashed bars, unstimulated PBMCs; dark gray bars, in absence of SOCS1-KIR pretreatment; light gray bars, in presence of SOCS1-KIR pretreatment. Dotted line indicates increase or decrease from unstimulated baseline. Statistical analysis = paired t-test to measure effect of SOCS1-KIR pretreatment; p-value listed above bars. Controls N = 9 horse samples; ERU N = 10 horse samples. LPS, lipopolysaccharide; ERU, equine recurrent uveitis; PBMCs, peripheral blood mononuclear cells.

Next, changes in cytokine responses to equine PBMCs by SOCS1-KIR that were stimulated with LPS were assessed. While preincubation of non-uveitic control PBMCs with SOCS1-KIR had no significant effect on LPS-driven interferon-gamma production, ERU PBMCs preincubated with SOCS1-KIR and stimulated with LPS (1 μg/mL) had significantly reduced levels of IFNγ compared to those lacking pretreatment [p = 0.04 (57% reduction)] (**Figure 3**). IP-10 levels were also reduced in ERU PBMCs pretreated with SOCS1-KIR [p-value = 0.004 (40% reduction)], which is consistent with evidence showing that IFNγ drives expression and production of IP-10 (63, 64). Under LPS stimulation, RANTES secretion was significantly reduced in ERU PBMCs only [p < 0.05 (57% reduction)] (**Figure 3**). Unlike the effects observed under PHA stimulation, SOCS1-KIR pretreatment did not affect TNFα or IL-10 secretion under LPS stimulation (**Figure 3**). Despite the modest induction of TNFα and IL-6 in LPS-stimulated ERU PBMCs, the two proinflammatory cytokines were positively correlated in ERU cells (p < 0.001, R = 0.90) (**Supplementary Table 3**). This positive induction has been shown previously in human monocytes, in which treatment with TNFα resulted in increased IL-6 secretion (65).

### SOCS1-KIR administration significantly increases IL-8 secretion in equine PBMCs

IL-8 is an important chemokine, as it relates to the inflammatory response, with a function in cell recruitment to sites of inflammation (66, 67). SOCS1-KIR administration promoted significant increases in IL-8 secretion in equine PBMCs that were independent of disease status and occurred in the presence of either PHA or LPS (**Figures 2**, **3**). Notably, SOCS1-KIR treatment increased secretion of IL-8 in the absence of stimulation as well (p = 0.0009 and 0.0144 in control and ERU PBMCs, respectively) (**Supplementary Tables 4**, **5**). In addition to IL-8 secretion, without mitogen stimulation, concentrations of IL-10, IL-6, and IL-1α were significantly increased in control equine PBMCs with SOCS1-KIR pretreatment (p-value = 0.0015, 0.0206, and 0.0199, respectively) (**Supplementary Table 4**). This effect was not observed in ERU PBMCs (**Supplementary Table 5**).

### SOCS1-KIR mimetic peptide is detectable in treated HeLa cells and equine PBMCs

**Figure 4:**
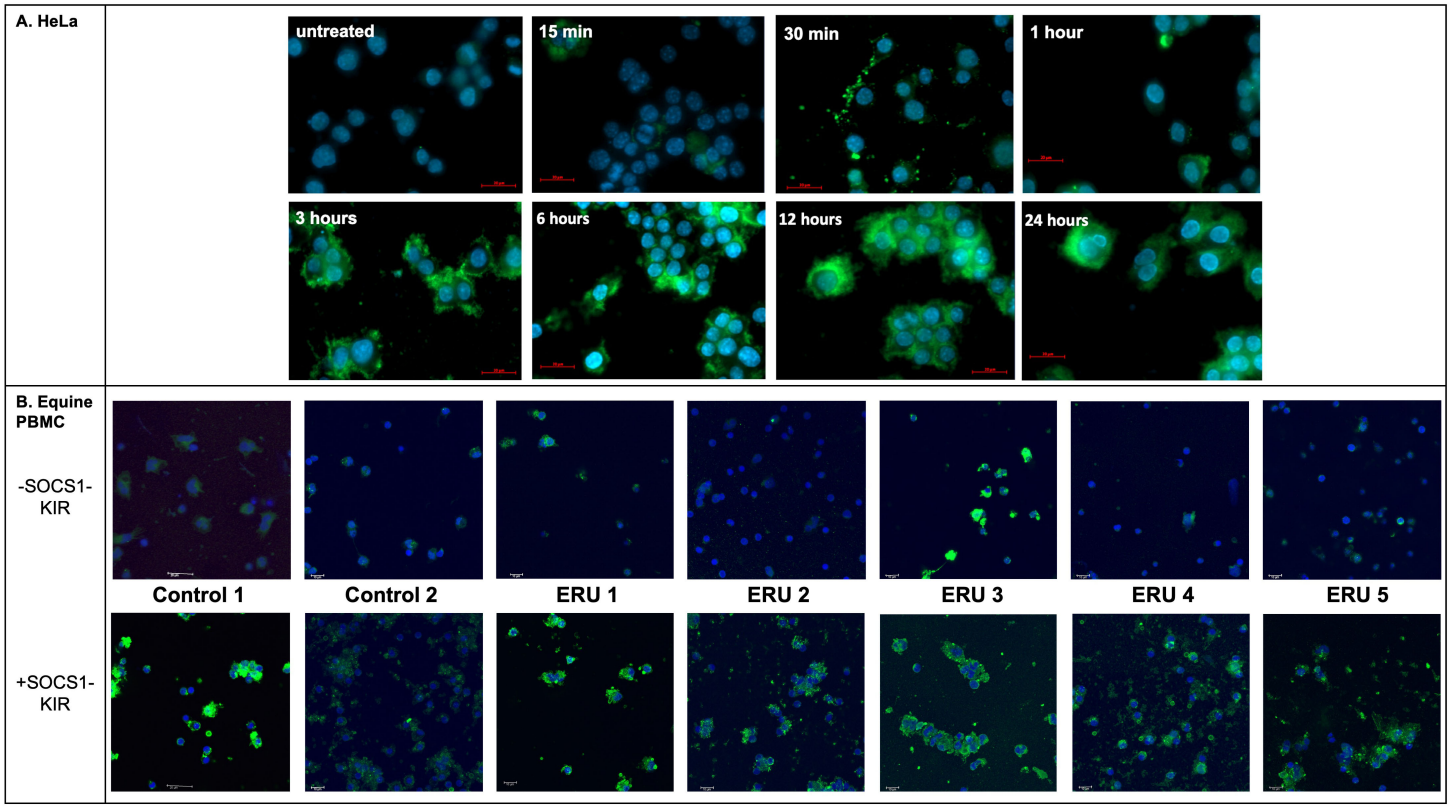
SOCS1-KIR is readily internalized in HeLa cells and equine PBMC isolated from ERU patients and controls *in vitro*. **(A)** HeLa cell line was incubated with SOCS1-KIR (33 µM) for the indicated time points, while **(B)** PBMCs from ERU patients (n= 5) or controls (n=2) were incubated for 24 hours, followed by immunofluorescent analysis. Images showing immunofluorescence of HeLa cells (N = 4 experimental replicates) (top) and equine PBMC (N = 7 biological replicates) (bottom) treated with SOCS1-KIR over 24 hours. Primary = anti-SOCS1-KIR, secondary = goat anti-rabbit IgG*AF488 (green), DAPI-stained nuclei (blue). Scale bars = 20 µm in HeLa and 10 µm in equine PBMC.

Factors contributing to proper drug design include demonstrated efficacy in treating disease, appropriate targeting of mechanisms thought to drive disease progression, and effective drug localization to tissue types affected by disease. Based on our previous studies showing that topical administration of a SOCS1-KIR mimetic mitigated clinical ERU symptoms (2) and our current studies showing that SOCS1-KIR mediated reductions in ERU-associated (58) TNFα in mitogen-activated equine PBMCs, we next sought to assess peptide localization in equine eyes treated with topical SOCS1-KIR. As a necessary first step, we evaluated peptide localization kinetics in HeLa cells treated with the SOCS1-KIR peptide at 33 μM SOCS1-KIR at varied times over a 24-hour period to better understand internalization kinetics followed by immunofluorescence analysis. While detection of DAPI-stained nuclei was visible at all time points in HeLa cells, the SOCS1-KIR peptide was only readily detectable 3 hours post-treatment. SOCS1-KIR was then detected in the HeLa cells for the duration of the 24-hour experiment (**Figure 4A**, **Supplementary Figure 6**). Having established an effective strategy to visualize internalized SOCS1-KIR in HeLa cells by immunofluorescence, we next validated the internalization of SOCS1-KIR in equine PBMCs treated with the peptide. Using peripheral blood isolated from seven horses, five with ERU and two controls, we incubated it with peptide for 24 hours, followed by peptide detection by indirect immunofluorescence. SOCS1-KIR was clearly visible in all sets of equine PBMCs after 24 hours of incubation (**Figure 4B**, **Supplementary Figure 7**).

### Topical administration of SOCS1-KIR mimetic was found to be safe in healthy horses

**Figure 5:**
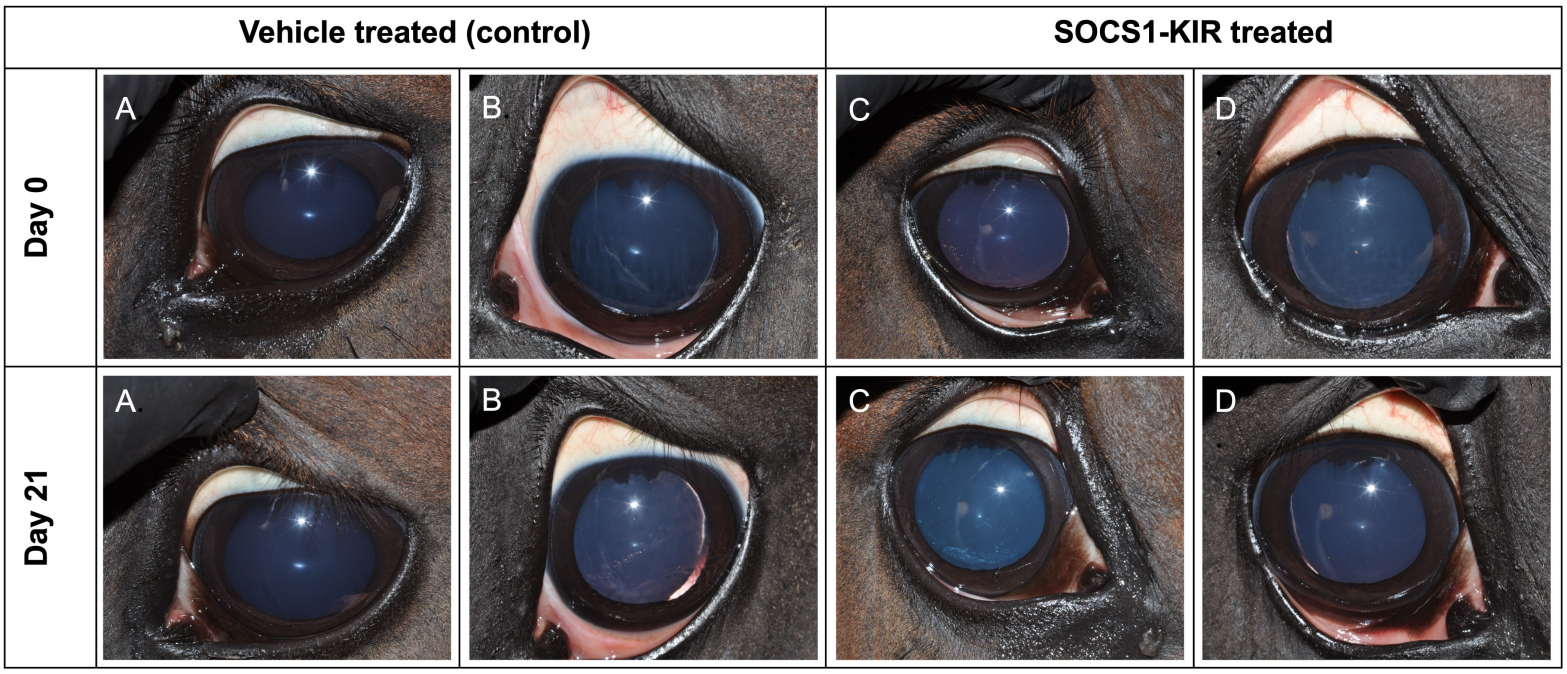
SOCS1-KIR mimetic treatment is safe for the equine eye. Equine eyes were treated topically with carrier (control) (left) or SOCS1-KIR (right) over 21 days. **(A, B)** Two eyes treated with carrier (controls). **(C, D)** Two eyes treated with SOCS1-KIR.

We previously demonstrated that the topical administration of a SOCS1-KIR mimetic (0.2 mg) to the eye of ERU horses reduced clinical symptoms in an open-label clinical trial (2). To better establish a safe working range for SOCS1-KIR mimetic treatment use, nine experimental, healthy horses were initially given complete ophthalmic examinations followed by ERG to establish baseline ocular profiles. ERG is a well-established method of assessing gross physiological changes in an intact retina (2). Physical examinations were also conducted. The horses were then treated with 0.2 mg, 1 mg, or 2 mg of SOCS1-KIR or carrier topically in one eye twice daily for 21 days. In addition, ERG and physical examinations were also performed on days 0, 7, 14, and 21 to measure any changes over the treatment period. Neither the physical examinations performed on days 7, 14, and 21 nor the ERG exams (measuring α waves, β waves, and flicker responses) revealed any distinctions from the initial examination (day 0) (**Supplementary Tables 6**, **7**). On days 0 and 21, eyes were imaged to assess anatomical change. As can be clearly seen, photographs taken on day 0 and day 21 were indistinct and indistinguishable from the photographs of eyes that received carrier (**Figure 5**). On day 21, after ERG and examinations, the experimental horses were euthanized, and eyes were enucleated for histopathologic and additional analyses. Histopathologic analysis revealed no distinction between SOCS1-KIR-treated and carrier-treated eyes. In summary, these data demonstrate that SOCS1-KIR was safely administered at a concentration of 2 mg, which was 10× higher than the previously used concentration, for 21 days.

### SOCS1-KIR is readily detectable in intravitreally injected equine retina

**Figure 6:**
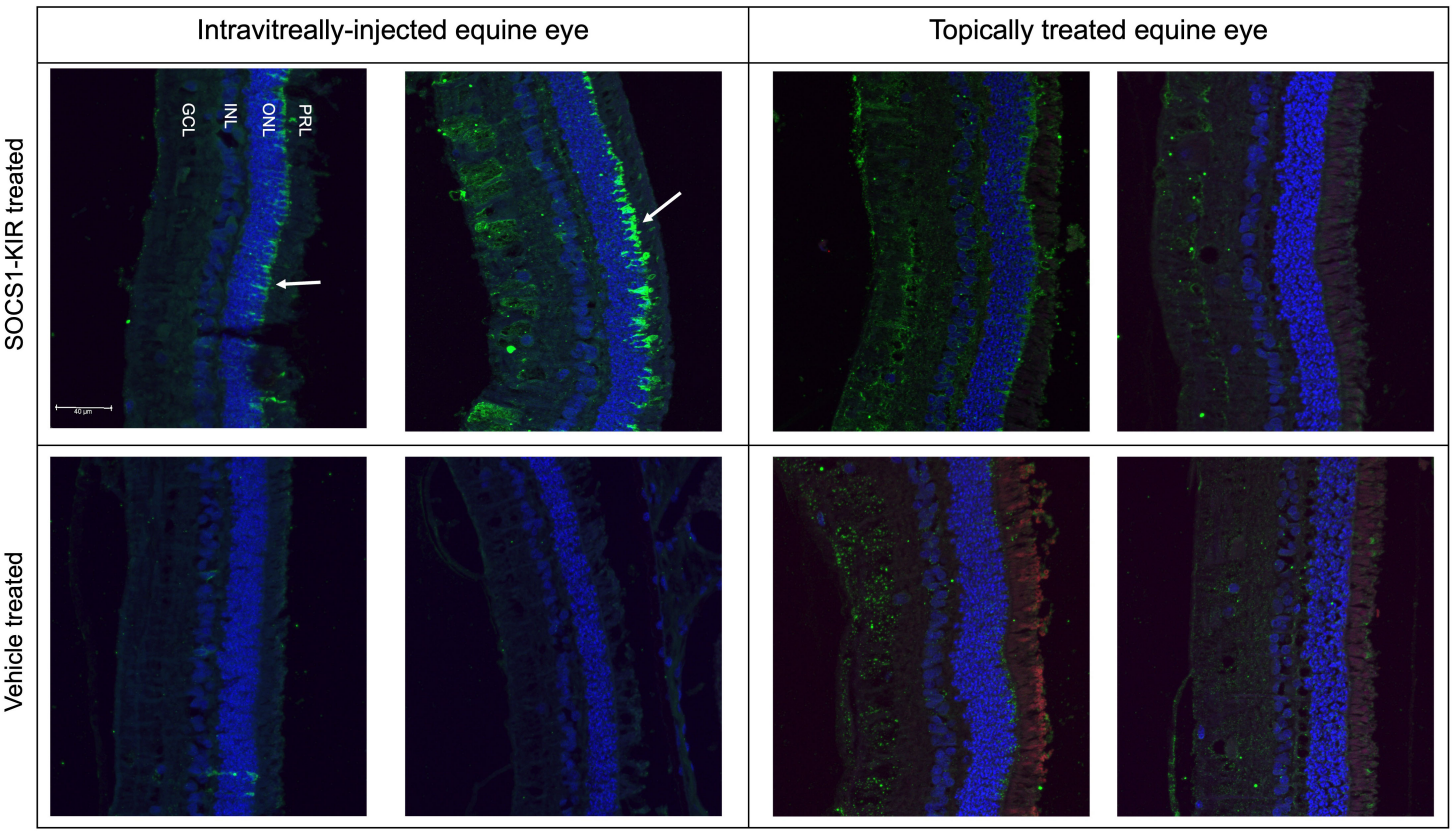
SOCS1-KIR mimetic peptide detectable in outer nuclear layer of intravitreally injected equine retina. Intravitreally injected SOCS1-KIR-treated retina (top left), intravitreally injected control retina (bottom left), topically administered SOCS1-KIR-treated retina (top right), and topically administered control retina (bottom right). Primary, anti-SOCS1-KIR; secondary, donkey anti-rabbit IgG*AF488 (green). Scale bar = 40 µm. GCL, ganglion cell layer; INL, inner nuclear layer; ONL, outer nuclear layer; PRL, photoreceptor layer.

Using the optimization results obtained during the detection of SOCS1-KIR by immunofluorescence in HeLa cells and equine PBMCs (**Figure 4**) *in vitro*, we next assessed the presence of the peptide in the enucleated eyes from our nine experimental horses, which were now paraffin-embedded. We also utilized the paraffin-embedded sections from our previous experiment, which included equine eyes from two horses that were injected intravitreally with 0.2 mg of SOCS1-KIR or carrier (2). The SOCS1-KIR mimetic peptide was readily detected in equine eyes when intravitreally injected and, as expected, was absent in eyes that received carrier (**Figure 6**). As denoted by the white arrow in **Figure 6**, intravitreally injected SOCS1-KIR was largely localized to the outer nuclear layer of the retina. We next evaluated the ocular structures of the ciliary body and retina obtained from the eyes of horses treated 21 days with either SOCS1-KIR twice daily or carrier. SOCS1-KIR was not detected in equine eyes that had been topically treated (**Figure 6**, **Supplementary Figure 8**).

### Topical SOCS1-KIR, administered as an eye drop, mitigates TNFα, IL-10, and IL-1α production in the eye of experimental horses

**Figure 7:**
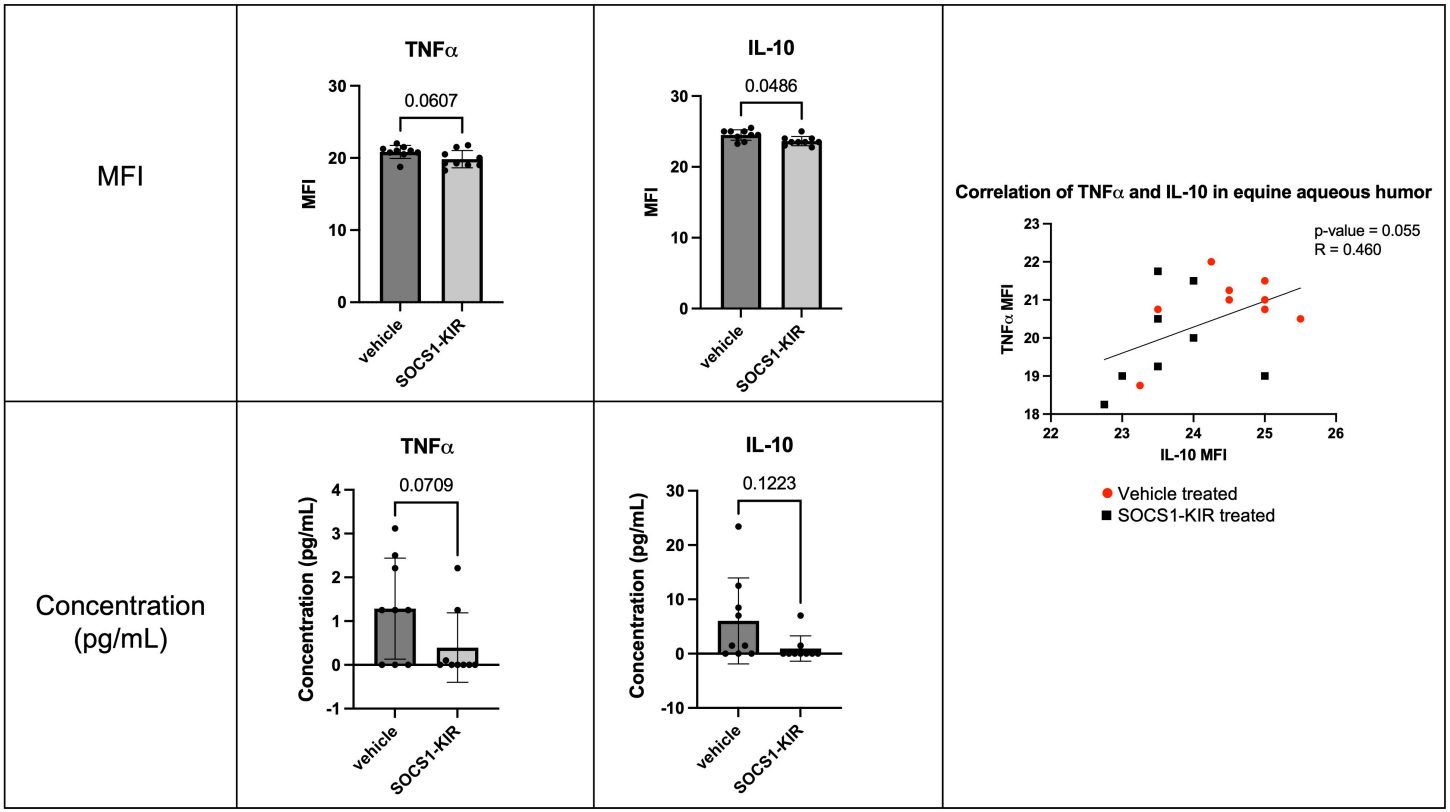
Topical administration of SOCS1-KIR mimetic reduces TNFα and IL-10 in equine aqueous humor. Cytokine MFI (top) and calculated protein concentration (bottom) of TNFα (left) and IL-10 (middle) in aqueous humor of healthy equine eyes treated topically with or without SOCS1-KIR mimetic peptide. MFI, mean fluorescence intensity of Luminex dataset. Each dot represents aqueous humor sample from an experimental equine eye. Bars indicate mean value + SD, statistical analyses = paired t-test and non-parametric Spearman’s correlations; vehicle-treated (red circle) and SOCS1-KIR-treated (black square) shown in correlation graph; linear regression model used to model correlation; p-value listed above bars. N = 18 horse eyes (nine SOCS1-KIR-treated, nine vehicle control).

Given that we have previously shown that topical administration of SOCS1-KIR to the equine eye mitigated clinical symptoms of disease in ERU horses, we hypothesized that topical administration of SOCS1-KIR would exert a measurable, mechanistic effect. To examine the immunomodulatory effects of SOCS1-KIR mimetic peptide treatment in the equine eye, we obtained aqueous and vitreous humor samples from the nine healthy horses treated with the SOCS1-KIR mimetic peptide or carrier for 3 weeks (twice daily). We then measured cytokine and chemokine levels present in the aqueous humor and vitreous humor using the MILLIPLEX Equine Cytokine/Chemokine Magnetic Bead Panel. Although overall cytokine concentrations were low in healthy equine aqueous humor and vitreous humor, as expected in the absence of overt disease, administration of SOCS1-KIR mimetic peptide treatment led to decreased levels of IL-10 and TNFα in equine aqueous humor when comparing SOCS1-KIR-treated eyes versus carrier control eyes (p-value = 0.0486 and 0.0607, respectively) (**Figure 7**). IL-10 and TNFα in aqueous humor were shown to be positively correlated, with lower concentrations of both cytokines observed in SOCS1-KIR-treated eyes (p-value = 0.055, R = 0.46) (**Figure 7**). We also assessed changes in chemokine levels in the equine eye but found no statistically significant differences. We observed no statistically significant differences in the vitreous humor in cytokine or chemokine levels.

**Figure 8:**
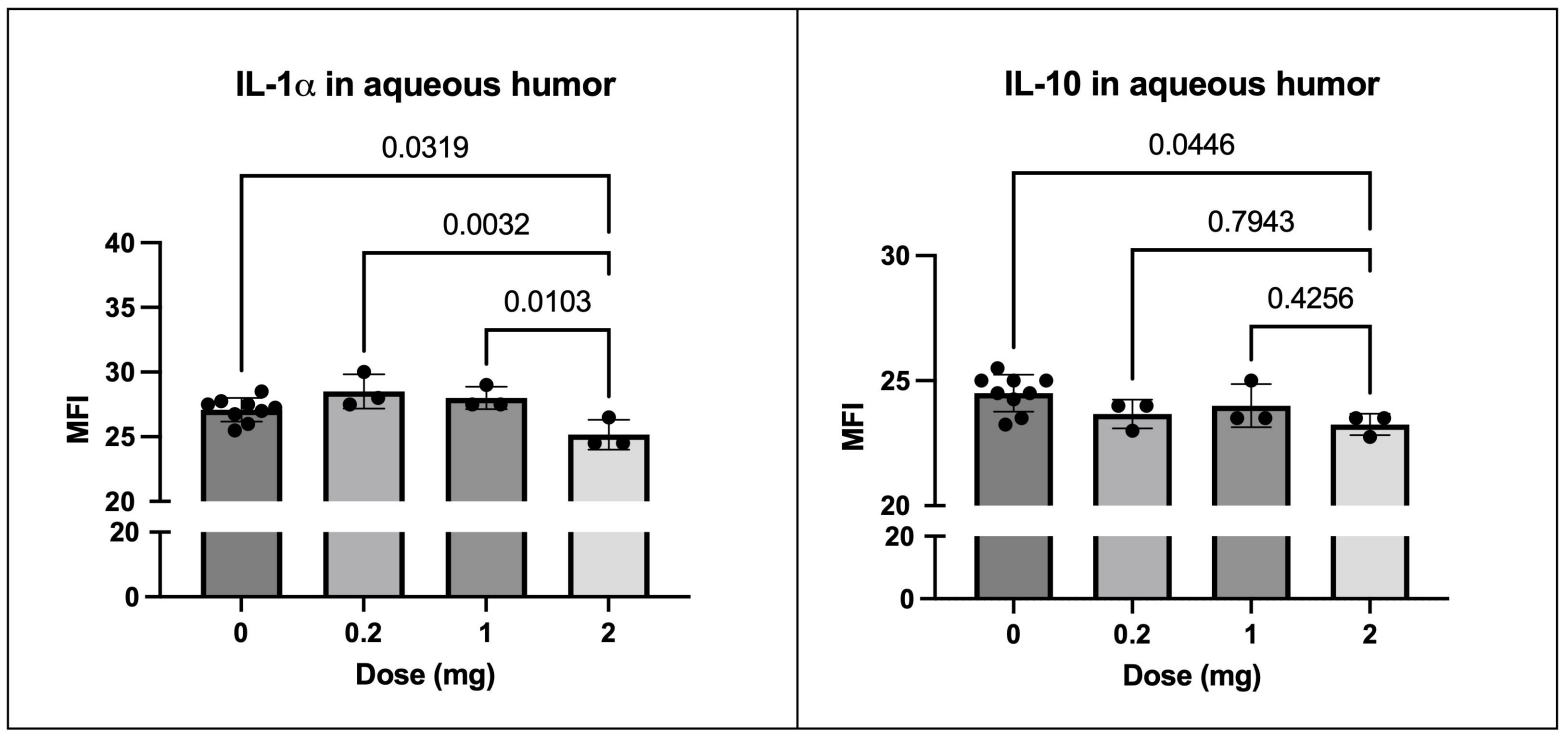
SOCS1-KIR treatment results in reduction of IL-1α and IL-10 in healthy equine aqueous humor. Cytokine MFI of IL-1α (left) comparing 2 mg vs. untreated (0), 0.2 mg, and 1 mg treatment. Cytokine MFI of IL-10 (right) comparing 2 mg vs. untreated (0), 0.2 mg, and 1 mg treatment. MFI, mean fluorescence intensity of Luminex dataset. Each dot represents aqueous humor sample from an experimental equine eye. Bars indicate mean value ± SD; statistical analysis = ordinary one-way ANOVA; p-value listed above bars.

As previously noted, we treated the horses with three different doses of SOCS1-KIR mimetic peptide. Although no treatment concentration presented with any overt pathology, we observed that IL-1α and IL-10 levels were significantly lower at the highest dose of SOCS1-KIR (2 mg) compared to vehicle-treated eyes (p-value = 0.03 and 0.04, respectively) (**Figure 8**).

## Discussion

Despite current standard-of-care treatments, recurrent uveitis remains a leading cause of sight decrement in both humans and horses. As such, there is an urgent need for novel options to treat recurrent uveitis patients who are intolerant to current strategies or with a disease that is refractory to the current standard of care. Our research group has chosen to focus on SOCS1, given its implicated natural role in the regulation of ocular inflammatory disease (68). We have previously shown that the topical administration of a peptide mimic of the SOCS1 kinase inhibitory region potently inhibited uveitis in both murine and rat models of induced disease, while also modulating the immune responses guiding those pathologies (7). Importantly, we have recently shown that the topical administration of SOCS1-KIR to the eye of equine patients afflicted with ERU was correlated to the amelioration of clinical symptoms (2). In this study, we assessed the ability of the SOCS1-KIR mimetic peptide to modulate immune responses induced by mitogenic stimulation of equine PBMCs isolated from both control and ERU horses. Additionally, we built upon our previous studies assessing the safety of topical SOCS1-KIR administration to the equine eye, evaluated the ocular localization of SOCS1-KIR peptide administered topically or intravitreally, and identified a biological signature for peptide administration in healthy equine eyes. We report here that SOCS1-KIR mimetic peptide treatment resulted in lowered PBMC cell proliferation in stimulated and unstimulated healthy equine PBMCs. SOCS1-KIR administration also led to significantly reduced TNFα and IL-10 secretion in PHA-stimulated equine PBMCs, isolated from both non-uveitis and ERU horses. Additionally, we observed a SOCS1-KIR-mediated reduction of IFNγ and subsequent IP-10 production in LPS-stimulated ERU PBMCs. Although SOCS1-KIR, which had been topically administered to the eye of healthy experimental horses, was undetectable, the administration resulted in significant reductions in TNFα and IL-10 in the aqueous humor. Together, these results demonstrate modulation of the equine immune system functions by SOCS1-KIR, building upon our previous studies demonstrating amelioration of ERU clinical symptoms by topical peptide administration.

While the proliferation of non-uveitic and ERU-derived PBMCs was statistically indistinct, our data suggest that the PBMCs were differentially responsive to mitogenic stimulation in terms of cytokine production. While the production of TNFα increased 34-fold in non-uveitic PBMCs after PHA mitogenic stimulation, TNFα production increased 49 times in uveitic PBMCs. Moreover, the fold increase of IL-6 (40-fold vs. 27-fold), IFNγ (~450-fold vs. ~90-fold), and RANTES (CCL5) (28-fold vs. 18-fold) was also higher in ERU cells upon PHA stimulation compared to controls. Our observed increases in these proinflammatory cytokines are consistent with previous reports showing increased stimulation responsiveness of PBMCs derived from uveitis patients compared to controls. Notably, one study showed that PBMCs from Behçet’s disease patients had higher levels of IFNγ and IL-6 secretion compared to controls upon interphotoreceptor retinoid-binding protein (IRBP) and S-antigen stimulation (69). Of the cytokines we evaluated, LPS measurably stimulated the production of IL-6, IL-8, and IL-10 in control PBMCs and the production of IL-8 and IL-10 in activated ERU PBMCs. As we used PBMCs (a mixed population of immune cells), we may have observed stimulation differences based on the types of cells typically activated by PHA or LPS. Like human PBMC populations, horses have ~70%–80% CD3^+^ T cells, with a 2:1 ratio of CD4^+^ to CD8^+^ and less than 20% B cells, with 5%–10% of PBMCs being monocytes/dendritic cells (70–73). That said, LPS may be more effective at stimulating a modest percentage of PBMCs (e.g., B cells and monocytes). Several works have shown that LPS significantly activates human monocyte cells, with pronounced increases in the secretion and expression of TNFα, IL-6, IL-8, and IL-10 (74, 75). While CD4^+^ T cells are the predominant TNFα-expressing lymphocyte, equine CD8^+^ T cells and B cells have displayed the ability to express TNFα upon PMA (76). Additional detailed studies critically evaluating the composition and differentiation of immune cells throughout the course of recurrent uveitis progression and using a combination of induced rodent models and peripheral blood from patients will likely provide further insight into disease mechanisms.

TNFα is an important proinflammatory cytokine, critical in the clearance of both pathogens and cancerous tissues. However, given its wide spectrum of cellular effects (cell survival, proliferation, programmed cell death, and necrosis) (77), aberrant TNFα signaling can also mediate damage in inflamed tissues, making the balance a delicate one. TNFα can be produced by numerous cell types of myeloid and lymphoid origins (78). Currently, TNFα blockers are used in several diseases, including human recurrent uveitis, as a therapeutic strategy to limit excessive TNFα signaling (79, 80). However, TNFα blockers are not efficacious in all patients, have been implicated in the susceptibility to some cancers, and may induce intolerable immunosuppression (81). Notably, SOCS1 is a critical regulator of not only TNFα production but also cellular responsiveness to the cytokine. It is well established that TNFα levels are elevated in rodent models of autoimmunity in the absence of SOCS1 (82). It was also demonstrated that while SOCS1^−/−^ mice experienced higher levels of TNFα, IL-10, and IL-17 compared to wild-type mice, in the context of viral infections, SOCS1 overexpression could decrease the concentrations of virally induced TNFα (83, 84). Additionally, SOCS1 can readily suppress TNFα-induced apoptosis, thus showing the ability of SOCS1 to regulate cellular responsiveness to existing TNFα (85, 86). While it is established that SOCS1 can inhibit the nuclear factor kappa-B (NF-κB) signaling cascade through E3-ubiquitin ligase-mediated degradation of its p65 subunit in the nucleus (87), we and others have previously shown that the kinase inhibitory region of SOCS1, administered as a peptide, can effectively mitigate TNFα production in both murine myeloid (48, 88) and lymphoid cells (46). Additionally, we have shown that SOCS1-KIR can limit cellular responsiveness to TNFα in murine models (89). However, evidence showing the capacity to translate these findings to relevant diseases (human or agricultural) is limited. In this manuscript, we show that SOCS1-KIR administration reduced TNFα production in PHA-activated PBMCs isolated from both control and ERU horses. We found that SOCS1-KIR mediated a 40% and 8% reduction in PHA-mediated TNFα production in control and ERU-derived PBMCs, respectively. Given that PHA (a T-cell mitogen) and not LPS (an APC mitogen) promoted TNFα production, we postulate that TNFα production by equine T lymphocytes is being targeted by SOCS1-KIR. This postulation is further supported by a previous study demonstrating the inhibition of TNFα by SOCS1-KIR in the mrl/lpr rodent model of lupus (46). To our knowledge, this is the first report showing that SOCS1-KIR mediated the mitigation of TNFα production in equine PBMCs. Although SOCS1-KIR effectively inhibited PHA-mediated TNFα in both healthy and ERU-derived PBMCs, the efficacy was strikingly approximately fivefold higher in healthy PBMCs compared to ERU. While we are unable to address the stark differences in efficacy between disease states, we predict that future experiments evaluating the intracellular production of TNFα in specific immune cell subsets (lymphoid and myeloid) will provide critical insight. These future studies will address our predictions that differences in the cellular composition of PBMCs derived from healthy and ERU horses contribute, at least in part, to differences in SOCS1-KIR responsiveness.

In the context of recurrent uveitis, TNFα is thought to disrupt the blood–retina barrier, promote proinflammatory immune cell influx into the eye, and play a critical role in disease progression (81). Under steady-state, healthy conditions, TNFα is thought to maintain immune surveillance and promote tissue maintenance in the eye (90–92). While TNFα is readily detected in the ocular tissues and aqueous humor of uveitis-affected eyes, the concentration is much lower in healthy equine eyes (58, 59, 61). Although we were unable to detect measurable levels of TNFα in the aqueous humor of our experimental horses using an equine TNFα ELISA kit, the increased sensitivity of the MILLIPLEX Equine Cytokine/Chemokine Magnetic Bead Panel allowed for the detection of low levels of TNFα in healthy horse aqueous humor. Significantly, we found that SOCS1-KIR treatment lowered TNFα concentration in the aqueous humor of healthy horses. Notably, IL-1α was significantly lower in the highest dose (2 mg/mL) of SOCS1-KIR treatment in equine aqueous humor compared to untreated controls and lower doses of SOCS1-KIR treatment, indicating a dose-dependent reduction in the equine eye. This result is consistent with previous studies showing the regulation of IL-1α by TNFα production and TNFα signal blockade (62, 93, 94). We feel that this finding provides a strong rationale for a multicenter trial evaluating the modulation of TNFα levels in the eyes of ERU horses receiving SOCS1-KIR treatment. Additionally, the work presented supports the hypothesis that regulation of T lymphocyte-specific TNFα production may be a mechanism by which reductions in ERU symptoms were previously observed (2).

IL-10 is produced by monocytes and lymphocytes following proinflammatory response (95). Treatment with IL-10 results in significantly lower expression and production of TNFα in PBMCs and alveolar macrophages (62). IL-10 has been shown in previous works to tightly regulate TNFα secretion through suppression of the NF-κB pathway (95, 96). In the work of Van der Poll et al., TNFα treatment resulted in a significant increase in IL-10 induction (97). These interactions indicate a potential feedback loop between these differing cytokines. We report a finding of decreased TNFα and IL-10 under SOCS1-KIR mimetic treatment. Particularly, in ERU PBMCs, we found a significant positive correlation between the two cytokines, with SOCS1-KIR appearing to significantly reduce both. Interestingly, the concentration of IL-10 is often significantly increased in the aqueous humor of uveitic eyes, as well as in systemic lupus erythematosus patients (58, 98). In this study, we observed a significant decrease in IL-10 concentration in the aqueous humor of SOCS1-KIR mimetic-treated healthy equine eyes. We hypothesize that increased IL-10 levels in patients are an effort by the immune system to counterbalance the inflammatory response and that the SOCS1-KIR-mediated reductions in IL-10 reflect an overall decrease in inflammatory signaling.

In our study, interferon-gamma production was not significantly reduced by SOCS1-KIR in PHA-stimulated PBMCs derived from ERU or healthy horses. However, SOCS1-KIR significantly reduced interferon gamma and IP-10 stimulation produced by LPS-stimulated, ERU-derived PBMCs. IP-10 is induced by IFNγ signaling by both T lymphocytes and antigen-presenting cells. IP-10 then acts primarily as a chemotactic factor for T lymphocytes and plays an important role in cellular proliferation (99). Our current data suggest that SOCS1-KIR had limited efficacy in directly mitigating IFNγ production in mitogen-stimulated T cells but could reduce IFNγ produced either by APC or by reducing an APC-derived factor necessary for T-cell production. The ability of SOCS1 to inhibit NF-κB signaling driven by TLR activation is well established (100–102). Additionally, it has been previously demonstrated that SOCS1-KIR administration inhibited interferon-gamma production in both murine T lymphocytes and monocytes (46, 88). The differential response observed between ERU and control PBMCs may again be indicative of the cell populations in the horses, or their differentiation states, allowing LPS-induced IFNγ secretion to be more tightly regulated by SOCS-KIR pretreatment in ERU PBMCs compared to controls. IFNγ activates Janus kinases, thus activating the JAK/STAT pathway and subsequent immune response (103). Our group and others (68) have shown that SOCS1-KIR administration results in a significant reduction of STAT1 phosphorylation and nuclear translocation in IFNγ- and IL-6-stimulated human and mouse cell lines (Sharma et al., unpublished) (43). As such, it may be possible that SOCS1-KIR administration inhibits both the induction of cytokines (such as IL-12) by LPS that induced IFNγ and subsequent IFNγ responsiveness. Regulated on activation, normal T cell expressed and secreted (RANTES or CCL5) is a well-known chemokine, which acts as a specific chemoattractant for Th1 cells (104, 105). RANTES has been implicated as a mediator of inflammation in both induced and spontaneous uveitis (57, 105). In this study, we observe a differential response between LPS-stimulated, SOCS1-KIR-treated control and uveitic equine PBMCs, with SOCS1-KIR mimetic administration significantly lowering RANTES secretion in ERU cells only. However, despite limitations, it is likely that SOCS1-KIR regulation of IFNγ, IP-10, and RANTES induced by LPS may have relevance in equine diseases.

IL-8 is a proinflammatory chemokine produced by monocytes, which acts as a chemotactic factor for T lymphocytes and granulocytes into sites of inflammation (66, 67, 106). IL-8 has also been shown to be important for mediating corneal wound healing (93). It is well established that IL-8, also known as CXCL-8, production can be guided by NF-κB programming initiated by IL-1 or TNFα signaling. Strikingly, our current studies show that SOCS1-KIR administration significantly and consistently increased IL-8 secretion in PBMCs isolated from both control horses and those with ERU. The SOCS1-KIR-mediated increases in IL-8 production occurred in the absence of overt stimulation and in the presence of mitogens LPS (TLR4) and PHA (T cells). The increased production, mediated by SOCS1-KIR, was despite SOCS1-KIR-mediated reductions in TNFα. Past works have investigated the role of IL-8 and type I IFN responses. Notably, IFNα appears to be a powerful inhibitor of IL-8 expression, and under viral infection conditions, IL-8 expression is significantly increased and, in turn, reduces the antiviral potency of IFNs (107–109). As previously discussed, SOCS1 is a potent inhibitor of type I IFN response, and it may be possible that the IL-8 increase we observed under SOCS1-KIR mimetic treatment is in response to IFNα signal inhibition (110). It has been recently shown that individuals presenting with STAT3 gain-of-function variants had downregulated IL-8 cellular expression levels (111). Notably, aqueous humor of uveitic eyes had higher concentrations of IL-8 than healthy controls, although we did not observe a significant increase in IL-8 in SOCS1 KIR mimetic-treated healthy equine eyes in our study. The critical role of SOCS protein in the inhibition of interferon-mediated STAT signaling, combined with these studies, may provide insight into the critical crosstalk with the STAT/SOCS/IL8 axis.

The translation of a therapeutic from an interesting experimental idea to a novel therapeutic is dependent upon safety, overall drug tolerability, and drug efficacy. We have previously shown that topical administration of SOCS1-KIR for 14 days was safe to the equine eye of healthy, control horses and had no observed negative impact on the overall health of the horses. SOCS1-KIR was also administered topically twice daily in three ERU horses for 8 months and was well tolerated. As a necessary next step in the evaluation of SOCS1-KIR as a novel therapeutic strategy in the treatment of ERU (and possibly non-infectious uveitis in humans), in this study, we evaluated the safety of a peptide dosing range and extended the evaluation time of the healthy control horses from 14 to 21 days. We found that the administration of a 10-fold higher concentration of SOCS1-KIR (compared to the dosing used in our previous report that showed efficacy) for a longer time (from 14 to 21 days) had no observed untoward effects in terms of either ocular or overall health of horses. Given that PBMCs obtained from ERU horses were more refractory to the reduction of PHA-mediated TNFα production than control, these results help to justify the use of higher SOCS1-KIR doses in ERU horses with more refractory disease. The use of healthy, experimental horses allowed us to assess potential drug localization in the eye and possible biological signatures. We found that the topical administration of SOCS1-KIR reduced TNFα and IL-10 at all administered doses and IL-1α at the highest dose. We believe these data demonstrating SOCS1-KIR-mediated reductions in TNFα, IL-10, and IL-1α could help guide the efficacy of an equine clinical trial through the safe sampling of equine ocular aqueous sampling over the duration of the treatment. Given that the KIR of SOCS1 can inhibit kinase activity without the SOCS box (7) and that its respective binding groove on JAK2 is highly conserved across several relevant mammalian species, including humans, mice, and horses (2), we predict that demonstrated efficacy in horses will be relevant to human disease. Consistent with our rationale, research is currently underway to examine the regulation of the Jak/STAT pathway in recurrent uveitis through the use of commercially available Jakinibs (112–118). Our data showing reductions in TNFα, IL-10, and IL1α by SOCS1-KIR also add to our previous study by providing a potential mechanism by which SOCS1-KIR mitigated ERU in our previous open-label clinical trial. Given that SOCS1-KIR regulates Janus kinases in a way that is distinct from current Jakinibs and that kinase regulation is distinct from steroidal mechanisms of action, we are hopeful to have increased options for the treatment of intractable uveitis in the future. Together, we feel that these results provide critical impetus and justification for a multicenter clinical trial evaluating SOCS1-KIR as a novel therapeutic for the treatment of ERU used as a monotherapy or combination therapy strategy.

This work underscores the immunomodulatory effects of topical SOCS1-KIR administration in the equine model, both *in vitro* and *in vivo*, although this study has limitations. Due to limited equine reagent availability, we used PBMCs instead of sorted cell populations. While our cohort of horses was reasonably age-matched, there was no exact breed matching with controls (i.e., all Appaloosas were affected with ERU in our study), which may play a role given the relevancy of genetic predispositions of disease in certain breeds. In our investigation of the effects of SOCS1-KIR administration, we did not specifically activate uveitogenic T lymphocytes, known to cause uveitic damage in the eye of uveitis patients; rather, we activated PBMCs non-specifically. However, by utilizing both PHA and LPS, which are known to differentially stimulate immune cells, we were able to investigate potential differences in response. Future studies will allow us to fully elucidate the differences in cell populations in ERU and healthy controls. This study focused on recurrent uveitis, particularly in horses, but there is evidence for translation between horses and humans in recurrent uveitis. Horses remain the best model for our study as equine-centered experimentation, but intensive examination of the safety and effects in human eyes would not be possible.

## Data Availability

The original contributions presented in the study are included in the article/**Supplementary Material**. Further inquiries can be directed to the corresponding author.

## Supplementary material

Supplementary Figure 1 Representative figure of paired t-test analysis of TNF secretion concentration of vehicle (left) vs SOCS1-KIR treated (right) PHA-stimulated equine PBMC. Each line-linked pair denotes cultured PBMC, isolated from an individual horse, in the presence of absence of SOCS1-KIR treatment. Mean of differences are shown to the right, with a significant reduction of TNFα in SOCS1-KIR treated samples.

Supplementary Figure 2 Effects of stimulation on cell proliferation of control and ERU PBMC. PBMC isolated from equine controls were stimulated with either 1, 10, and 100 µg/mL PHA or LPS for 72 hours. Cell proliferation of stimulated samples was compared to unstimulated cells using ordinary one-way ANOVA, p-values are listed above bars. Control N = 17 horse samples; ERU N = 10 horse samples.

Supplementary Figure 3 Effects of stimulation on control PBMC cytokine and chemokine secretion. PBMC isolated from equine controls were stimulated with either 1 or 100 µg/mL PHA, or 1 µg/mL LPS for 72 hours. Concentrations of cytokines and chemokines were compared to unstimulated cells using ordinary one-way ANOVA, p-values are listed above bars. N = 13 control horse samples.

Supplementary Figure 4 Effects of stimulation on ERU PBMC cytokine and chemokine secretion. PBMC isolated from horses with ERU were stimulated with either 1 or 100 µg/mL PHA, or 1 µg/mL LPS for 72 hours. Concentrations of cytokines and chemokines were compared to unstimulated cells using ordinary one-way ANOVA, p-value is listed above bars. N = 10 ERU horse samples.

Supplementary Figure 5 TNFα and IL-10 secretions are positively correlated under PHA stimulation in ERU PBMC. Spearman correlations between log10-transformed TNFα and IL-10 secretions under PHA stimulation in ERU (left) and control (right) PBMC with (black squares) and without (red circles) SOCS1-KIR mimetic peptide pretreatment. Each dot represents an individual horse. Statistical analysis = nonparametric Spearman correlations; linear regression model used to model correlation; Control N = 13 horse samples; ERU N = 10 horse samples.

Supplementary Figure 6 SOCS1-KIR is statistically significantly detectable in SOCS1-KIR treated HeLa cells after 3 hours. SOCS1-KIR detection over 24 hours of treatment in HeLa cells. Statistical analysis = ordinary one-way ANOVA, p-value is listed above bars. N = 4 technical replicates.

Supplementary Figure 7 SOCS1-KIR fluorescent quantification in equine PBMC after 24 hours treatment. Statistical analysis = paired, T-test, p-value is listed above bar. N = 7 biological replicates.

Supplementary Figure 8 SOCS1-KIR mimetic peptide is undetectable in equine ciliary body. Intravitreally injected SOCS1-KIR treated ciliary body **(A)**, intravitreally-injected carrier (control) ciliary body **(B)**, topically administered SOCS1-KIR treated ciliary body **(C)**, topically administered control ciliary body **(D)**. Primary: anti-SOCS1-KIR, secondary: donkey anti-rabbit IgG (AF488). Scale bar= 50 µm.
