# Supplementary material for: A peptide mimic of SOCS1 modulates equine peripheral immune cells in vitro and ocular effector functions in vivo: implications for recurrent uveitis
Source: Front Immunol. 2025 Jan 10;15:1513157. doi: 10.3389/fimmu.2024.1513157 (PMC11757128; doi:10.3389/fimmu.2024.1513157)

Supplementary Figures

Stafford et al. 2024 ERU

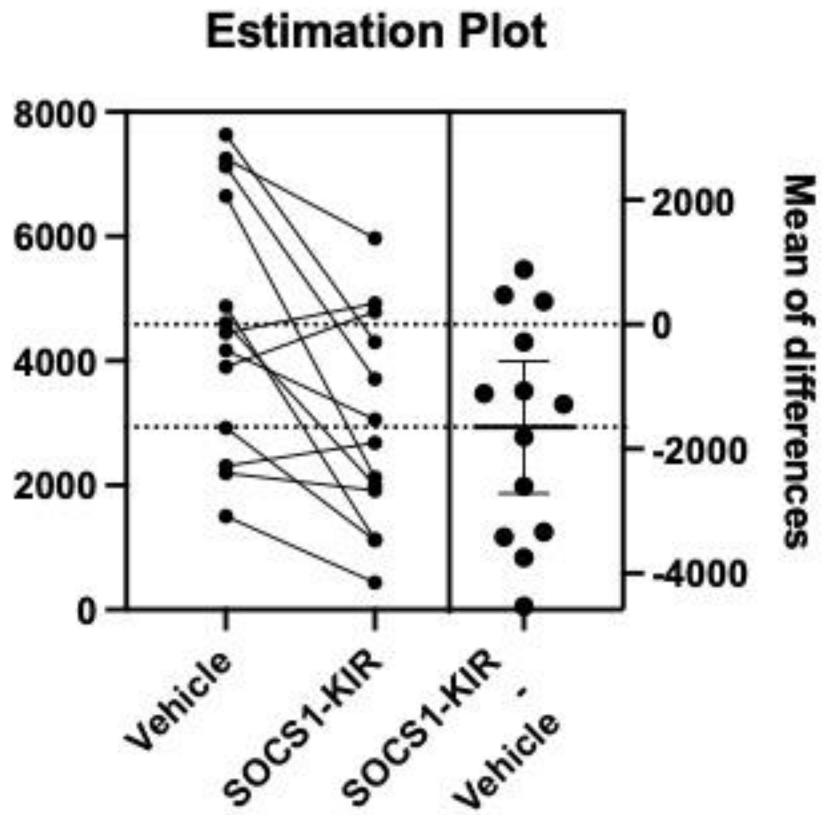
Estimation Plot
8000
6000
4000
2000
0
2000
0
-2000
-4000
Mean of differences
Vehicle
SOCS1-KIR
SOCS1-KIR - Vehicle

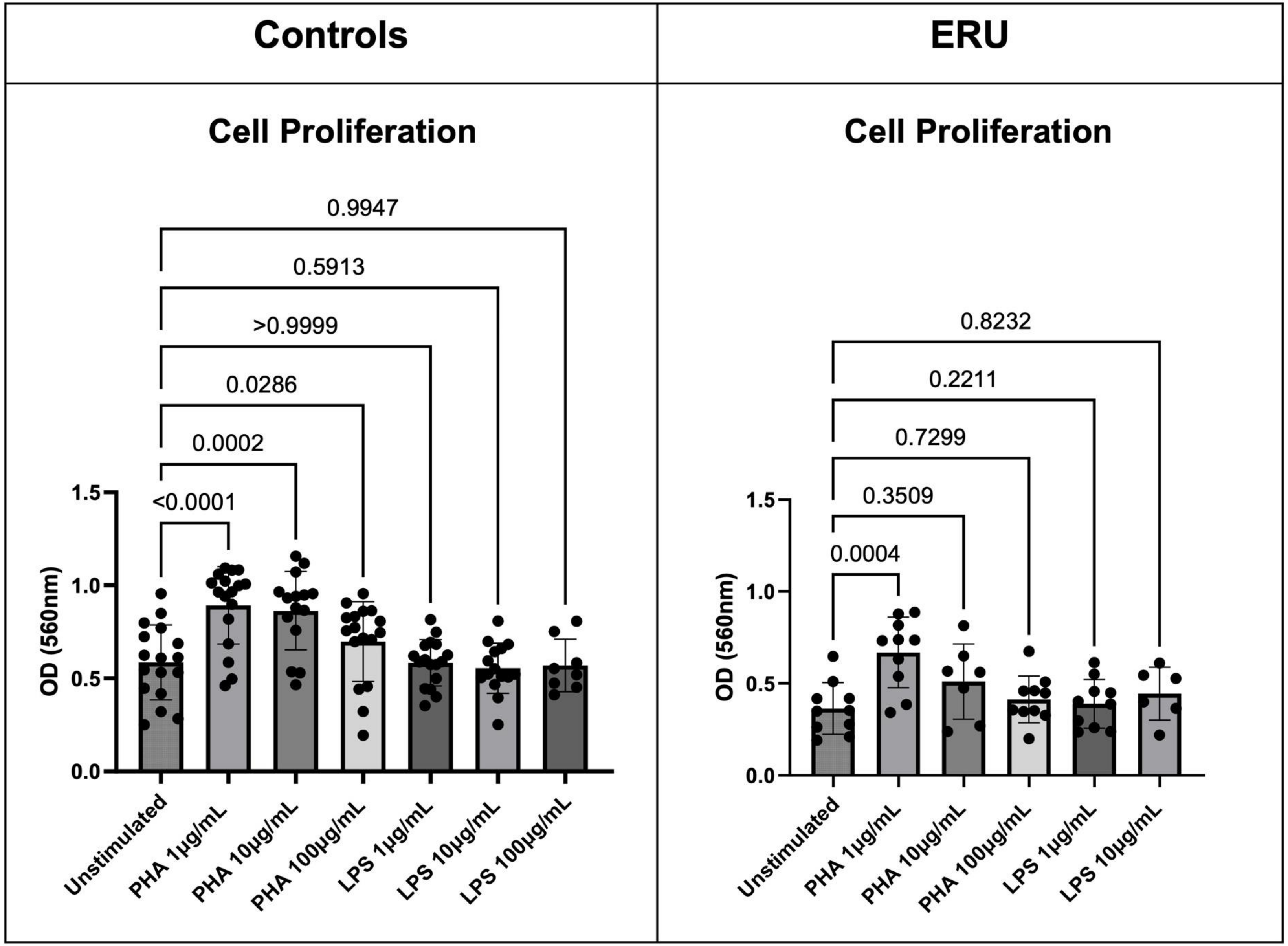
Controls
ERU
Cell Proliferation
0.9947
0.5913
>0.9999
0.0286
0.0002
<0.0001
OD (560nm)
1.5
1.0
0.5
0.0
Unstimulated
PHA 1µg/mL
PHA 10µg/mL
PHA 100µg/mL
LPS 1µg/mL
LPS 10µg/mL
LPS 100µg/mL
Cell Proliferation
0.8232
0.2211
0.7299
0.3509
0.0004
OD (560nm)
1.5
1.0
0.5
0.0
Unstimulated
PHA 1µg/mL
PHA 10µg/mL
PHA 100µg/mL
LPS 1µg/mL
LPS 10µg/mL

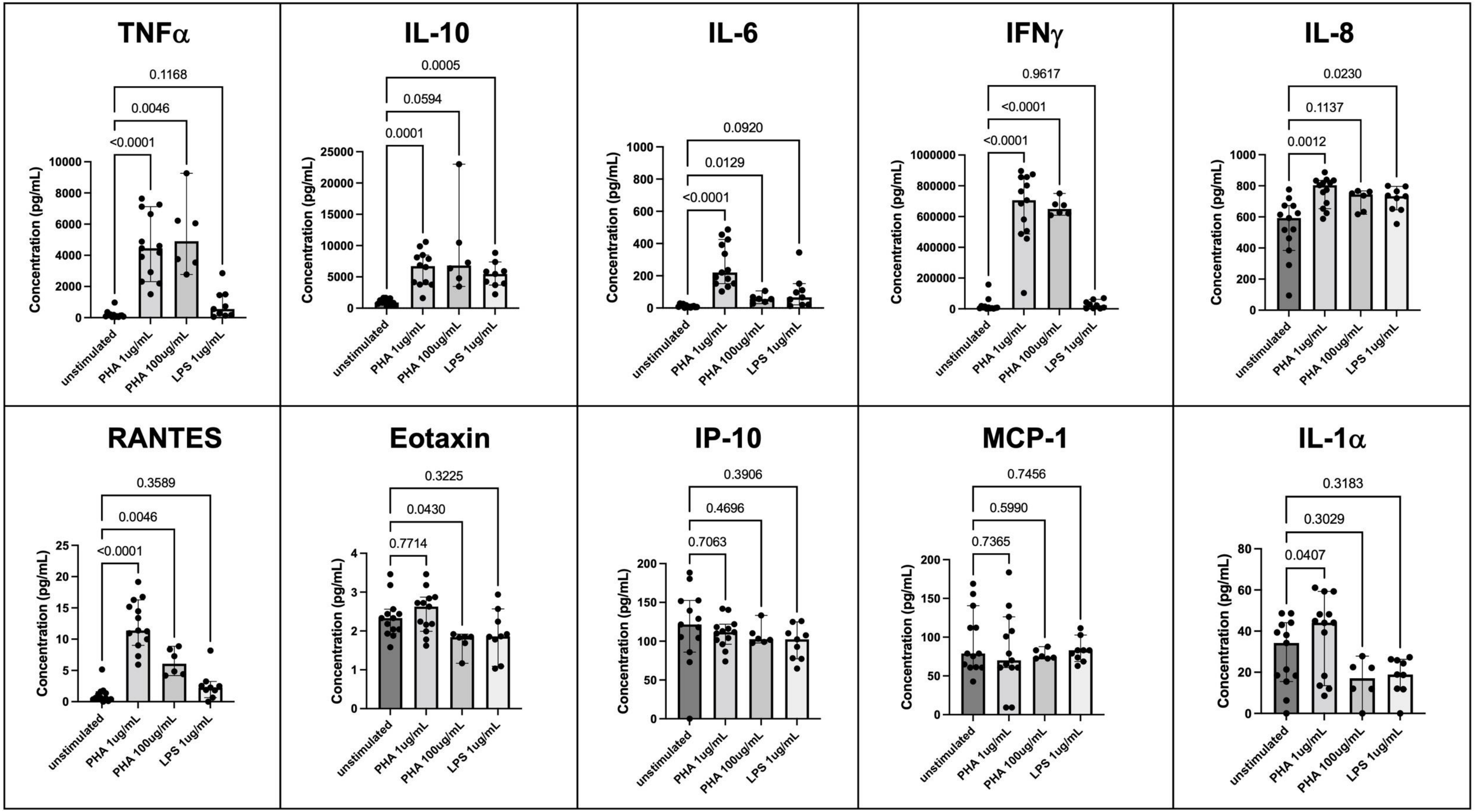
TNFα
0.1168
0.0046
<0.0001
Concentration (pg/mL)
10000
8000
6000
4000
2000
0
unstimulated
PHA 1ug/mL
PHA 100ug/mL
LPS 1ug/mL
IL-10
0.0005
0.0594
0.0001
25000
20000
15000
10000
5000
0
IL-6
0.0920
0.0129
<0.0001
1000
800
600
400
200
0
IFNγ
0.9617
<0.0001
<0.0001
1000000
800000
600000
400000
200000
0
IL-8
0.0230
0.1137
0.0012
1000
800
600
400
200
0
RANTES
0.3589
0.0046
<0.0001
25
20
15
10
5
0
Eotaxin
0.3225
0.0430
0.7714
4
3
2
1
0
IP-10
0.3906
0.4696
0.7063
200
150
100
50
0
MCP-1
0.7456
0.5990
0.7365
200
150
100
50
0
IL-1α
0.3183
0.3029
0.0407
80
60
40
20
0

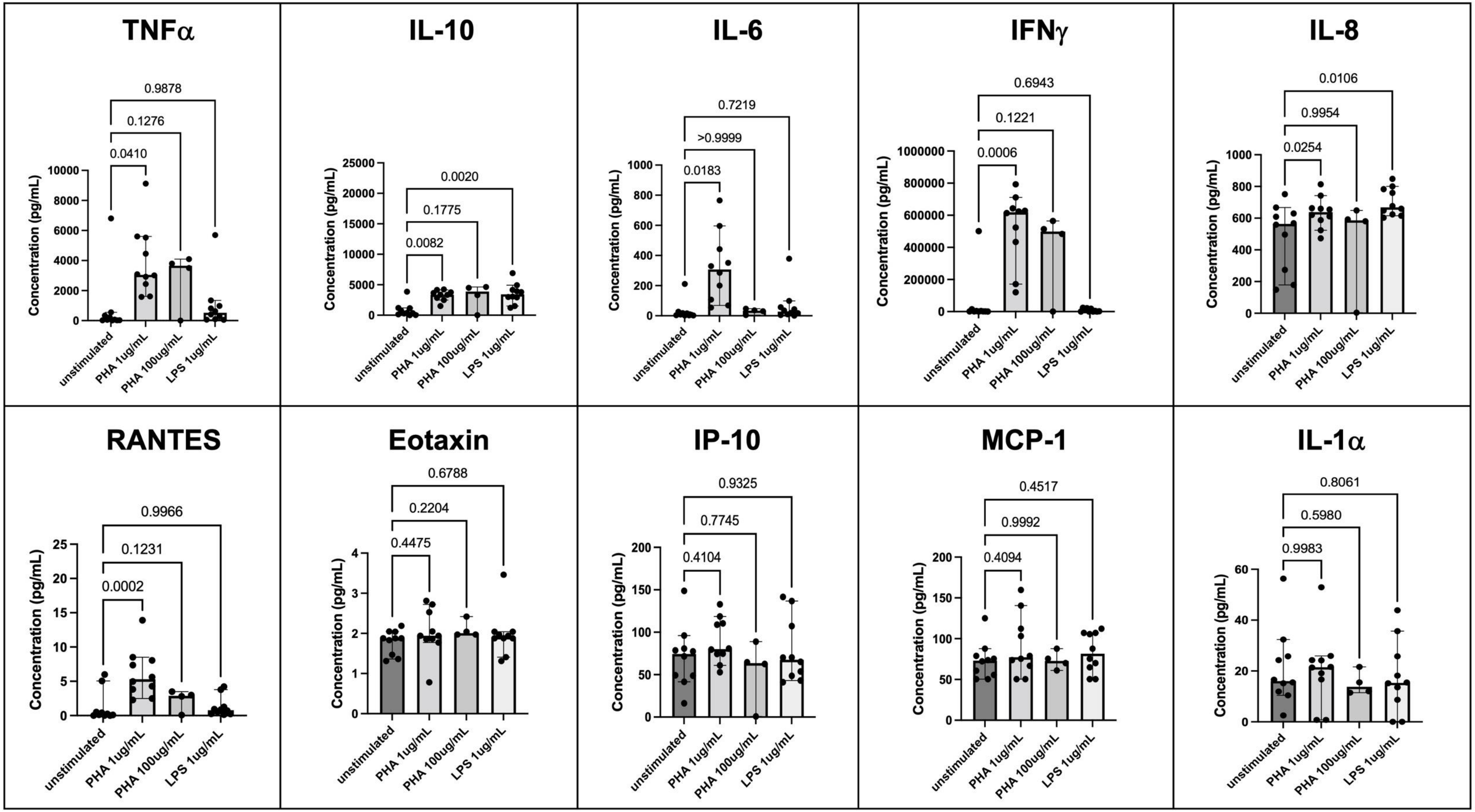
TNFα
0.9878
0.1276
0.0410
IL-10
0.0020
0.1775
0.0082
IL-6
0.7219
>0.9999
0.0183
IFNγ
0.6943
0.1221
0.0006
IL-8
0.0106
0.9954
0.0254
RANTES
0.9966
0.1231
0.0002
Eotaxin
0.6788
0.2204
0.4475
IP-10
0.9325
0.7745
0.4104
MCP-1
0.4517
0.9992
0.4094
IL-1α
0.8061
0.5980
0.9983
Concentration (pg/mL)
unstimulated
PHA 1ug/mL
PHA 100ug/mL
LPS 1ug/mL

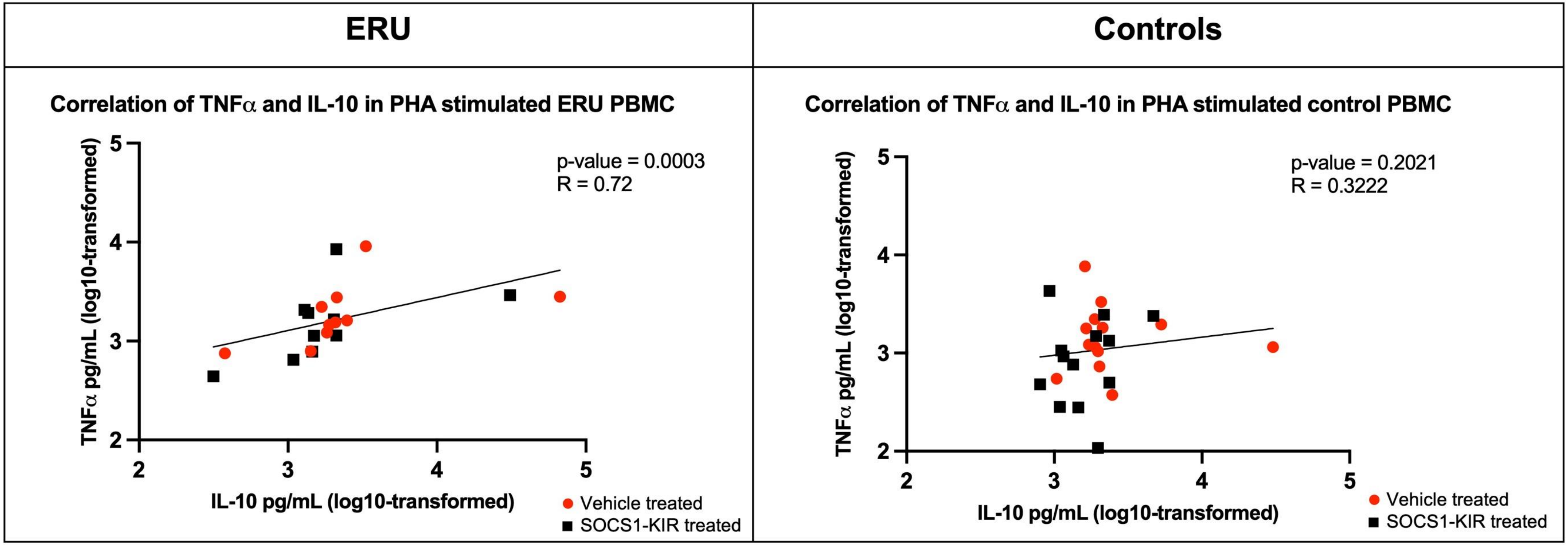
ERU
Controls
Correlation of TNFα and IL-10 in PHA stimulated ERU PBMC
p-value = 0.0003
R = 0.72
TNFα pg/mL (log10-transformed)
IL-10 pg/mL (log10-transformed)
Vehicle treated
SOCS1-KIR treated
Correlation of TNFα and IL-10 in PHA stimulated control PBMC
p-value = 0.2021
R = 0.3222
TNFα pg/mL (log10-transformed)
IL-10 pg/mL (log10-transformed)
Vehicle treated
SOCS1-KIR treated

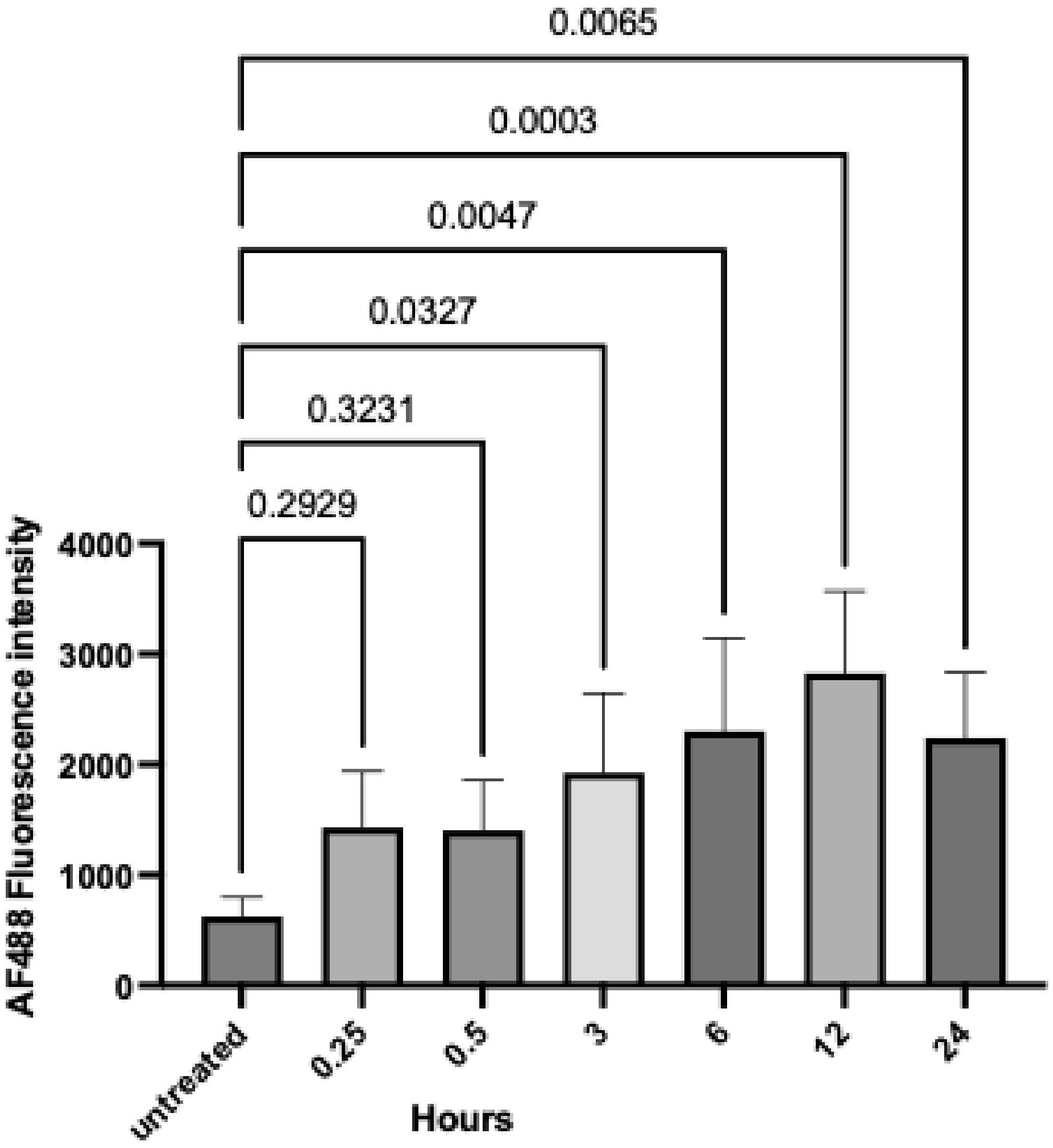
0.0065
0.0003
0.0047
0.0327
0.3231
0.2929
AF488 Fluorescence intensity
4000
3000
2000
1000
0
untreated
0.25
0.5
3
6
12
24
Hours

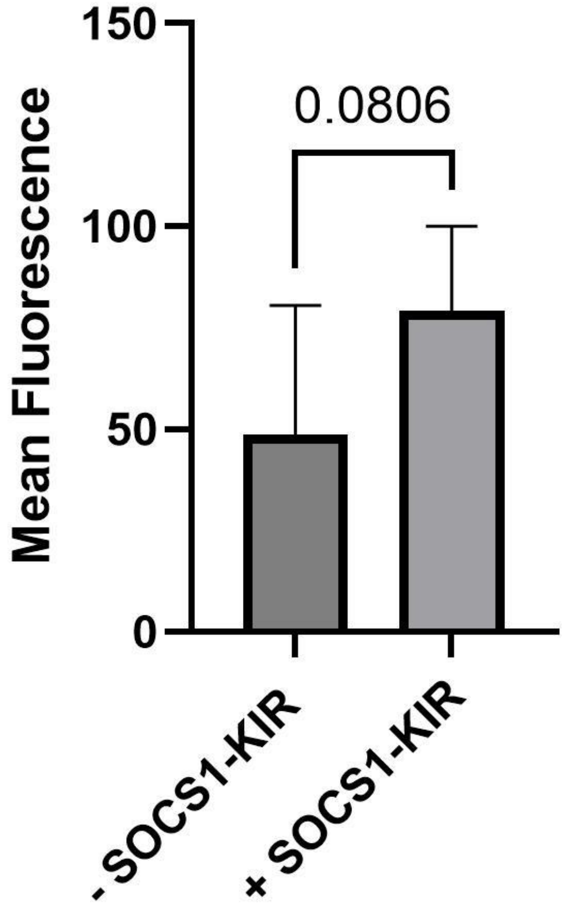
Mean Fluorescence
150
100
50
0
0.0806
- SOCS1-KIR
+ SOCS1-KIR

Intravitreally-injected equine eye	Topically-treated equine eye

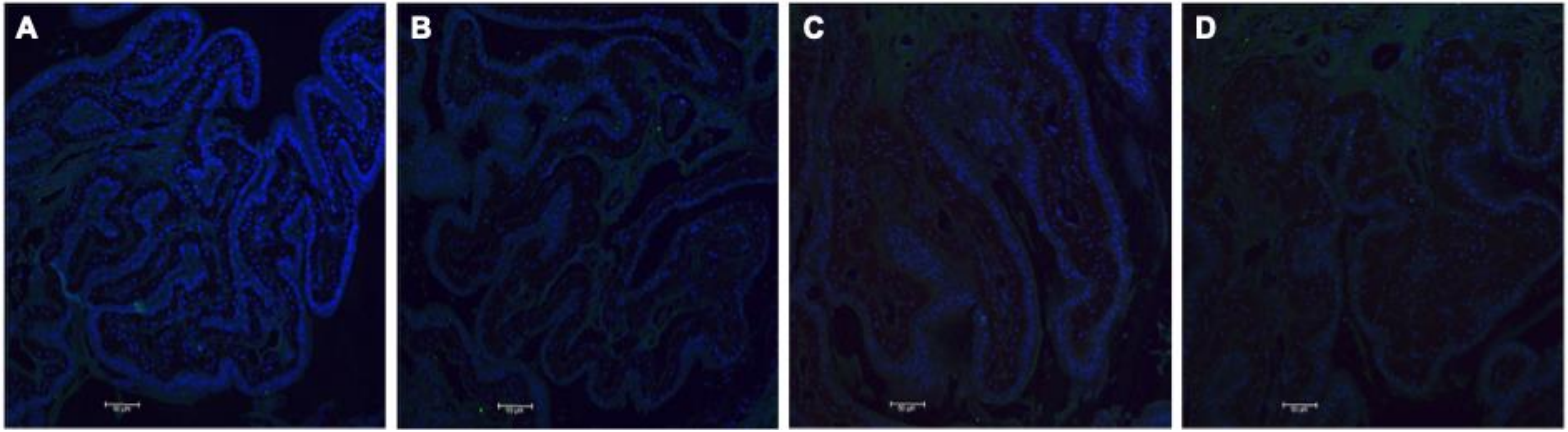

Supplement: Supplementary Figure 1 — Representative figure of paired t-test analysis of TNF secretion concentration of vehicle (left) vs SOCS1-KIR treated (right) PHA-stimulated equine PBMC. Each line-linked pair denotes cultured PBMC, isolated from an individual horse, in the presence of absence of SOCS1-KIR treatment. Mean of differences are shown to the right, with a significant reduction of TNFα in SOCS1-KIR treated samples. [file DataSheet1.pdf]
