# Supplementary material for: A peptide mimic of SOCS1 modulates equine peripheral immune cells in vitro and ocular effector functions in vivo: implications for recurrent uveitis
Source: Front Immunol. 2025 Jan 10;15:1513157. doi: 10.3389/fimmu.2024.1513157 (PMC11757128; doi:10.3389/fimmu.2024.1513157)
Supplement: Supplementary file 2 [file Table1.docx]

Supplementary Table 1.

| p-values | Eotaxin | TNFα | IL-6 | IL-10 | IFNγ | IL-8 | RANTES | IP-10 | MCP-1 |
| --- | --- | --- | --- | --- | --- | --- | --- | --- | --- |
| Eotaxin |  | 0.003 | 0.06 | 0.74 | 0.001 | 0.009 | 0.15 | 0.007 | 0.001 |
| TNFα | 0.003 |  | 0.002 | 0.72 | 0.00002 | 0.036 | 0.001 | 0.001 | 0.004 |
| IL-6 | 0.06 | 0.002 |  | 0.70 | 0.002 | 0.22 | 0.027 | 0.07 | 0.13 |
| IL-10 | 0.74 | 0.72 | 0.70 |  | 0.96 | 0.63 | 0.71 | 0.30 | 0.41 |
| IFNγ | 0.001 | 0.00002 | 0.002 | 0.96 |  | 0.014 | 0.012 | 0.001 | 0.009 |
| IL-8 | 0.009 | 0.036 | 0.22 | 0.63 | 0.014 |  | 0.13 | 0.015 | 0.019 |
| RANTES | 0.15 | 0.001 | 0.027 | 0.71 | 0.012 | 0.13 |  | 0.003 | 0.017 |
| IP-10 | 0.007 | 0.001 | 0.07 | 0.30 | 0.001 | 0.015 | 0.003 |  | 0.002 |
| MCP-1 | 0.001 | 0.004 | 0.13 | 0.41 | 0.009 | 0.019 | 0.017 | 0.002 |  |
| IL-1α | 0.98 | 0.57 | 0.10 | 0.95 | 0.80 | 0.46 | 0.83 | 0.77 | 0.55 |

| R | Eotaxin | TNFa | IL-6 | IL-10 | IFNg | IL-8 | RANTES | IP-10 | MCP-1 |
| --- | --- | --- | --- | --- | --- | --- | --- | --- | --- |
| Eotaxin |  | 0.78 | 0.54 | -0.10 | 0.83 | 0.71 | 0.42 | 0.72 | 0.80 |
| TNFa | 0.78 |  | 0.79 | -0.11 | 0.93 | 0.59 | 0.83 | 0.84 | 0.75 |
| IL-6 | 0.54 | 0.79 |  | 0.12 | 0.80 | 0.37 | 0.62 | 0.52 | 0.44 |
| IL-10 | -0.10 | -0.11 | 0.12 |  | 0.02 | -0.15 | -0.12 | -0.31 | -0.25 |
| IFNg | 0.83 | 0.93 | 0.80 | 0.02 |  | 0.68 | 0.69 | 0.84 | 0.71 |
| IL-8 | 0.71 | 0.59 | 0.37 | -0.15 | 0.68 |  | 0.45 | 0.67 | 0.65 |
| RANTES | 0.42 | 0.83 | 0.62 | -0.12 | 0.69 | 0.45 |  | 0.77 | 0.66 |
| IP-10 | 0.72 | 0.84 | 0.52 | -0.31 | 0.84 | 0.67 | 0.77 |  | 0.80 |
| MCP-1 | 0.80 | 0.75 | 0.44 | -0.25 | 0.71 | 0.65 | 0.66 | 0.80 |  |
| IL-1a | 0.01 | 0.17 | 0.48 | -0.02 | 0.08 | -0.23 | 0.07 | -0.09 | 0.18 |

Supplementary Table 1. Spearman correlations of secreted cytokines and chemokines of PHA 1μg/mL stimulated control PBMC. N = 13 control horses.

Supplementary Table 2.

| p-values | Eotaxin | TNFα | IL-6 | IL-10 | IFNγ | IL-8 | RANTES | IP-10 | MCP-1 |
| --- | --- | --- | --- | --- | --- | --- | --- | --- | --- |
| Eotaxin |  | 0.10 | 0.12 | 0.07 | 0.003 | 0.12 | 0.16 | 0.039 | 0.22 |
| TNFα | 0.10 |  | 0.007 | 0.004 | 0.06 | 0.39 | 0.027 | 0.06 | 0.22 |
| IL-6 | 0.12 | 0.007 |  | 0.006 | 0.17 | 0.07 | 0.020 | 0.39 | 0.76 |
| IL-10 | 0.07 | 0.004 | 0.006 |  | 0.07 | 0.22 | 0.10 | 0.06 | 0.58 |
| IFNγ | 0.003 | 0.06 | 0.17 | 0.07 |  | 0.37 | 0.10 | 0.003 | 0.05 |
| IL-8 | 0.12 | 0.39 | 0.07 | 0.22 | 0.37 |  | 0.49 | 0.49 | 0.97 |
| RANTES | 0.16 | 0.027 | 0.020 | 0.10 | 0.10 | 0.49 |  | 0.33 | 0.61 |
| IP-10 | 0.039 | 0.06 | 0.39 | 0.06 | 0.003 | 0.49 | 0.33 |  | 0.05 |
| MCP-1 | 0.22 | 0.22 | 0.76 | 0.58 | 0.05 | 0.97 | 0.61 | 0.05 |  |
| IL-1α | 0.22 | 0.48 | 0.82 | 0.86 | 0.06 | 0.53 | 0.11 | 0.07 | 0.21 |

| R | Eotaxin | TNFα | IL-6 | IL-10 | IFNγ | IL-8 | RANTES | IP-10 | MCP-1 |
| --- | --- | --- | --- | --- | --- | --- | --- | --- | --- |
| Eotaxin | 1.00 | 0.55 | 0.53 | 0.61 | 0.85 | 0.53 | 0.49 | 0.67 | 0.43 |
| TNFα | 0.55 | 1.00 | 0.81 | 0.84 | 0.62 | 0.31 | 0.71 | 0.62 | 0.43 |
| IL-6 | 0.53 | 0.81 | 1.00 | 0.82 | 0.48 | 0.61 | 0.73 | 0.31 | 0.12 |
| IL-10 | 0.61 | 0.84 | 0.82 | 1.00 | 0.60 | 0.43 | 0.55 | 0.62 | 0.20 |
| IFNγ | 0.85 | 0.62 | 0.48 | 0.60 | 1.00 | 0.32 | 0.56 | 0.85 | 0.64 |
| IL-8 | 0.53 | 0.31 | 0.61 | 0.43 | 0.32 | 1.00 | 0.25 | 0.25 | -0.02 |
| RANTES | 0.49 | 0.71 | 0.73 | 0.55 | 0.56 | 0.25 | 1.00 | 0.35 | 0.19 |
| IP-10 | 0.67 | 0.62 | 0.31 | 0.62 | 0.85 | 0.25 | 0.35 | 1.00 | 0.64 |
| MCP-1 | 0.43 | 0.43 | 0.12 | 0.20 | 0.64 | -0.02 | 0.19 | 0.64 | 1.00 |
| IL-1α | 0.42 | 0.25 | 0.09 | 0.07 | 0.63 | 0.22 | 0.54 | 0.61 | 0.43 |

Supplementary Table 2. Spearman correlations of secreted cytokines and chemokines of PHA 1μg/mL stimulated ERU PBMC. N = 10 ERU horses.

Supplementary Table 3.

| p-values | Eotaxin | TNFα | IL-6 | IL-10 | IFNγ | IL-8 | RANTES | IP-10 | MCP-1 |
| --- | --- | --- | --- | --- | --- | --- | --- | --- | --- |
| Eotaxin |  | 0.65 | 0.60 | 0.31 | 0.68 | 0.031 | 0.97 | 0.024 | 0.13 |
| TNFα | 0.65 |  | 0.001 | 0.49 | 0.68 | 0.92 | 0.044 | 0.73 | 0.52 |
| IL-6 | 0.60 | 0.001 |  | 0.45 | 0.31 | 0.97 | 0.06 | 0.89 | 0.37 |
| IL-10 | 0.31 | 0.49 | 0.45 |  | 0.95 | 0.017 | 0.97 | 0.23 | 0.78 |
| IFNγ | 0.68 | 0.68 | 0.31 | 0.95 |  | 0.26 | 0.15 | 0.13 | 0.12 |
| IL-8 | 0.031 | 0.92 | 0.97 | 0.017 | 0.26 |  | 0.95 | 0.07 | 0.62 |
| RANTES | 0.97 | 0.044 | 0.06 | 0.97 | 0.15 | 0.95 |  | 0.81 | 0.62 |
| IP-10 | 0.024 | 0.73 | 0.89 | 0.23 | 0.13 | 0.07 | 0.81 |  | 0.90 |
| MCP-1 | 0.13 | 0.52 | 0.37 | 0.78 | 0.12 | 0.62 | 0.62 | 0.90 |  |

|  | Eotaxin | TNFα | IL-6 | IL-10 | IFNγ | IL-8 | RANTES | IP-10 | MCP-1 |
| --- | --- | --- | --- | --- | --- | --- | --- | --- | --- |
| Eotaxin |  | -0.17 | -0.19 | 0.36 | 0.15 | 0.69 | 0.02 | 0.72 | 0.51 |
| TNFα | -0.17 |  | 0.90 | 0.25 | 0.15 | -0.04 | 0.66 | -0.13 | -0.23 |
| IL-6 | -0.19 | 0.90 |  | 0.27 | 0.36 | 0.02 | 0.62 | -0.05 | -0.32 |
| IL-10 | 0.36 | 0.25 | 0.27 |  | 0.03 | 0.75 | -0.02 | 0.42 | 0.10 |
| IFNγ | 0.15 | 0.15 | 0.36 | 0.03 |  | 0.39 | 0.49 | 0.52 | -0.52 |
| IL-8 | 0.69 | -0.04 | 0.02 | 0.75 | 0.39 |  | 0.03 | 0.60 | 0.18 |
| RANTES | 0.02 | 0.66 | 0.62 | -0.02 | 0.49 | 0.03 |  | 0.09 | -0.18 |
| IP-10 | 0.72 | -0.13 | -0.05 | 0.42 | 0.52 | 0.60 | 0.09 |  | -0.05 |
| MCP-1 | 0.51 | -0.23 | -0.32 | 0.10 | -0.52 | 0.18 | -0.18 | -0.05 |  |
| IL-1a | 0.46 | -0.32 | -0.41 | 0.09 | 0.05 | 0.37 | 0.31 | 0.22 | 0.40 |

Supplementary Table 3. Spearman correlations of secreted cytokines and chemokines of LPS 1μg/mL stimulated ERU PBMC. N = 10 ERU horses.

| **Supplementary Table 4.** | **-SOCS1-KIR** | **+SOCS1-KIR** | **-SOCS1-KIR** | **+SOCS1-KIR** | **N=13** |
| --- | --- | --- | --- | --- | --- |
| Control unstimulated | MEAN + SD | | MEDIAN (IQR1,IQR3) | | p-value (paired T-test) |
| Eotaxin | 2.33 +/- 0.52 | 2.81 +/- 0.73 | 2.33 [2.04,2.44] | 2.84 [2.26,3.52] | 0.0594 |
| TNFα | 197.19 +/- 247.63 | 136.26 +/- 106.31 | 125.6 [74.73,182.18] | 80.46 [58.84,223.53] | 0.34 |
| **IL-6** | **11.11 +/- 8.27** | **17.43 +/- 8.61** | **8 [5.53,15.49]** | **17.59 [10.22,23.64]** | **0.0208** |
| **IL-10** | **1026.69 +/- 463.74** | **1609.30 +/- 572.54** | **916.25 [703.31,1455.1]** | **1432.95 [1205.08,1825.76]** | **0.0015** |
| IFNγ | 23068.70 +/- 43739.86 | 12906.19 +/- 15032.80 | 7655.27 [3951.21,15444.9] | 7132.41 [3018.01,13963.43] | 0.829 |
| **IL-8** | **533.83 +/- 189.25** | **730.03 +/- 147.85** | **593.08 [462.11,670.89]** | **764.64 [636.38,829.09]** | **0.0009** |
| RANTES | 1.10 +/- 1.34 | 1.71 +/- 1.07 | 0.6 [0.41,1.36] | 1.59 [0.81,2.31] | 0.1366 |
| IP-10 | 119.21 +/- 49.22 | 158.76 +/- 82.63 | 121.57 [104.23,151.74] | 165.28 [104.94,218.81] | 0.064 |
| MCP-1 | 93.32 +/- 40.60 | 73.55 +/- 32.95 | 78.99 [61.31,112.22] | 66.80 [60.73,86.20] | 0.0763 |
| **IL-1α** | **28.93 +/- 16.41** | **39.94 +/- 19.05** | **34.23 [18.34,43.02]** | **36.14 [26.42,53.55]** | **0.0199** |

Supplementary Table 4. Mean and median of cytokines and chemokines of unstimulated control PBMC with and without SOCS1-KIR pretreatment. N = 13 control horses; statistical analysis = paired T-tests.

| **Supplementary Table 5.** | **-SOCS1-KIR** | **+SOCS1-KIR** | **-SOCS1-KIR** | **+SOCS1-KIR** | **N=10** |
| --- | --- | --- | --- | --- | --- |
| ERU unstimulated | MEAN + SD | | MEDIAN (IQR1,IQR3) | | p-value (paired T-test) |
| Eotaxin | 1.79 +/- 0.30 | 2.29 +/- 0.56 | 1.88 [1.56,2.0] | 2.19 [2.04,2.35] | 0.058 |
| TNFα | 811.19 +/- 2112.07 | 144.12 +/- 185.94 | 60.53 [22.44,305.52] | 77.92 [31.30,133.04] | 0.3511 |
| IL-6 | 29.81 +/- 64.42 | 14.60 +/- 10.55 | 7.55 [2.85,17.56] | 11.92 [9.51,13.19] | 0.4842 |
| IL-10 | 813.26 +/- 1155.85 | 755.1 +/- 763.66 | 365.73 [169.36,1057.24] | 421.185 [159.02,1165.49] | 0.8381 |
| IFNγ | 52876.25 +/- 157699.19 | 6587.39 +/- 7853.61 | 1366.93 [844.74,3959.26] | 4498.09 [809.7,8697.26] | 0.727 |
| **IL-8** | **488.17 +/- 211.84** | **597.03 +/- 142.14** | **563.34 [329.29,624.30]** | **653.29 [563.63,684.86]** | **0.0144** |
| RANTES | 1.24 +/- 2.27 | 1.10 +/- 1.71 | 0.18 [0.04,0.49] | 0.30 [0.06,1.31] | 0.8195 |
| IP-10 | 70.89 +/- 36.02 | 73.48 +/- 44.93 | 74.44 [48.90,80.15] | 70.09 [63.44,86.17] | 0.8071 |
| MCP-1 | 73.05 +/- 22.22 | 72.22 +/- 31.20 | 73.07 [56.85,78.08] | 67.68 [61.27,71.78] | 0.9553 |
| IL-1α | 21.08 +/- 15.02 | 24.38 +/- 17.68 | 16.04 [12.73,25.41] | 22.53 [16.34,28.76] | 0.5214 |

Supplementary Table 5. Mean and median of cytokines and chemokines of unstimulated ERU PBMC with and without SOCS1-KIR pretreatment. N = 10 ERU horses; statistical analysis = paired T-tests.

| **1 mg dose (n=3)** | **Day 0** | | **Day 1** | | **Day 7** | | **Day 14** | | **Day 21** | |
| --- | --- | --- | --- | --- | --- | --- | --- | --- | --- | --- |
| **Ocular observation variables** | **SOCS1** | **CTRL** | **SOCS1** | **CTRL** | **SOCS1** | **CTRL** | **SOCS1** | **CTRL** | **SOCS1** | **CTRL** |
| **Conjunctival Discharge** | 0.0 | 0.0 | 0.0 | 0.0 | 0.0 | 0.0 | 0.0 | 0.0 | 0.0 | 0.0 |
| **Conjunctival Congestion/Hyperemia** | 0.0 | 0.0 | 0.0 | 0.0 | 0.0 | 0.0 | 0.3 | 0.0 | 0.0 | 0.0 |
| **Conjunctival Swelling** | 0.0 | 0.0 | 0.0 | 0.0 | 0.0 | 0.0 | 0.0 | 0.0 | 0.0 | 0.0 |
| **Cornea** | 0.0 | 0.0 | 0.0 | 0.0 | 0.0 | 0.0 | 0.3 | 0.0 | 0.0 | 0.0 |
| **Surface Area of Cornea Involvement** | 0.0 | 0.0 | 0.0 | 0.0 | 0.0 | 0.0 | 0.3 | 0.0 | 0.0 | 0.0 |
| **Vascularization of Cornea** | 0.0 | 0.0 | 0.0 | 0.0 | 0.0 | 0.0 | 0.0 | 0.0 | 0.0 | 0.0 |
| **Menace Response** | + | + | + | + | + | + | + | + | + | + |
| **Pupillary Response** | +/+ | +/+ | +/+ | +/+ | +/+ | +/+ | +/+ | +/+ | +/+ | +/+ |
| **Vertical Pupil Diameter** |  |  |  |  |  |  |  |  |  |  |
| **Aqueous Flare** | 0.0 | 0.0 | 0.0 | 0.0 | 0.0 | 0.0 | 0.0 | 0.0 | 0.0 | 0.0 |
| **Cellular Flare** | 0.0 | 0.0 | 0.0 | 0.0 | 0.0 | 0.0 | 0.0 | 0.0 | 0.0 | 0.0 |
| **Hyphema/Hypopyon** | 0.0 | 0.0 | 0.0 | 0.0 | 0.0 | 0.0 | 0.0 | 0.0 | 0.0 | 0.0 |
| **Iris Involvement** | 0.0 | 0.0 | 0.0 | 0.0 | 0.0 | 0.0 | 0.0 | 0.0 | 0.0 | 0.0 |
| **Lens – Cataract Location** | 0.3 | 0.0 | 0.3 | 0.0 | 0.3 | 0.0 | 0.3 | 0.0 | 0.3 | 0.0 |
| **Lens – Cataract Stage** | 0.3 | 0.0 | 0.3 | 0.0 | 0.3 | 0.0 | 0.3 | 0.0 | 0.3 | 0.0 |
| **Vitreous** | 0.0 | 0.0 | 0.0 | 0.0 | 0.0 | 0.0 | 0.0 | 0.0 | 0.0 | 0.0 |
| **Vitreal Hemorrhage** | 0.0 | 0.0 | 0.0 | 0.0 | 0.0 | 0.0 | 0.0 | 0.0 | 0.0 | 0.0 |
| **Retinal Detachment – Type** | 0.0 | 0.0 | 0.0 | 0.0 | 0.0 | 0.0 | 0.0 | 0.0 | 0.0 | 0.0 |
| **Retinal Detachment – Area** | 0.0 | 0.0 | 0.0 | 0.0 | 0.0 | 0.0 | 0.0 | 0.0 | 0.0 | 0.0 |
| **Retinal Hemorrhage** | 0.0 | 0.0 | 0.0 | 0.0 | 0.0 | 0.0 | 0.0 | 0.0 | 0.0 | 0.0 |
| **Choroidal/Retinal Inflammation** | 0.0 | 0.0 | 0.0 | 0.0 | 0.0 | 0.0 | 0.0 | 0.0 | 0.0 | 0.0 |
| **Fluorescein** | 0.0 | 0.0 | 0.0 | 0.0 | 0.0 | 0.0 | 0.3 | 0.0 | 0.0 | 0.0 |
| **IOP** | 16.3 | 18.7 | 17.0 | 15.3 | 15.7 | 15.7 | 15.3 | 16.0 | 17.7 | 18.0 |
| **Ocular Discomfort – Description** | 0.0 | 0.0 | 0.0 | 0.0 | 0.0 | 0.0 | 0.0 | 0.0 | 0.0 | 0.0 |
| **Ocular Discomfort - Duration** | 0.0 | 0.0 | 0.0 | 0.0 | 0.0 | 0.0 | 0.0 | 0.0 | 0.0 | 0.0 |

| **2 mg dose (n=3)** | **Day 0** | | **Day 1** | | **Day 7** | | **Day 14** | | **Day 21** | |
| --- | --- | --- | --- | --- | --- | --- | --- | --- | --- | --- |
| **Ocular observation variables** | **SOCS1** | **CTRL** | **SOCS1** | **CTRL** | **SOCS1** | **CTRL** | **SOCS1** | **CTRL** | **SOCS1** | **CTRL** |
| **Conjunctival Discharge** | 0.0 | 0.0 | 0.0 | 0.0 | 0.0 | 0.0 | 0.0 | 0.0 | 0.0 | 0.0 |
| **Conjunctival Congestion/Hyperemia** | 0.0 | 0.0 | 0.0 | 0.0 | 0.0 | 0.0 | 0.3 | 0.0 | 0.0 | 0.3 |
| **Conjunctival Swelling** | 0.0 | 0.0 | 0.0 | 0.0 | 0.0 | 0.0 | 0.0 | 0.0 | 0.0 | 0.0 |
| **Cornea** | 0.0 | 0.0 | 0.0 | 0.0 | 0.0 | 0.0 | 0.0 | 0.0 | 0.0 | 0.0 |
| **Surface Area of Cornea Involvement** | 0.0 | 0.0 | 0.0 | 0.0 | 0.0 | 0.0 | 0.0 | 0.0 | 0.0 | 0.0 |
| **Vascularization of Cornea** | 0.0 | 0.0 | 0.0 | 0.0 | 0.0 | 0.0 | 0.0 | 0.0 | 0.0 | 0.0 |
| **Menace Response** | + | + | + | + | + | + | + | + | + | + |
| **Pupillary Response** | +/+ | +/+ | +/+ | +/+ | +/+ | +/+ | +/+ | +/+ | +/+ | +/+ |
| **Vertical Pupil Diameter** |  |  |  |  |  |  |  |  |  |  |
| **Aqueous Flare** | 0.0 | 0.0 | 0.0 | 0.0 | 0.0 | 0.0 | 0.0 | 0.0 | 0.0 | 0.0 |
| **Cellular Flare** | 0.0 | 0.0 | 0.0 | 0.0 | 0.0 | 0.0 | 0.0 | 0.0 | 0.0 | 0.0 |
| **Hyphema/Hypopyon** | 0.0 | 0.0 | 0.0 | 0.0 | 0.0 | 0.0 | 0.0 | 0.0 | 0.0 | 0.0 |
| **Iris Involvement** | 0.0 | 0.0 | 0.0 | 0.0 | 0.0 | 0.0 | 0.0 | 0.0 | 0.0 | 0.0 |
| **Lens – Cataract Location** | 0.0 | 0.0 | 0.0 | 0.0 | 0.0 | 0.0 | 0.0 | 0.0 | 0.0 | 0.0 |
| **Lens – Cataract Stage** | 0.0 | 0.0 | 0.0 | 0.0 | 0.0 | 0.0 | 0.0 | 0.0 | 0.0 | 0.0 |
| **Vitreous** | 0.0 | 0.0 | 0.0 | 0.0 | 0.0 | 0.0 | 0.0 | 0.0 | 0.0 | 0.0 |
| **Vitreal Hemorrhage** | 0.0 | 0.0 | 0.0 | 0.0 | 0.0 | 0.0 | 0.0 | 0.0 | 0.0 | 0.0 |
| **Retinal Detachment – Type** | 0.0 | 0.0 | 0.0 | 0.0 | 0.0 | 0.0 | 0.0 | 0.0 | 0.0 | 0.0 |
| **Retinal Detachment – Area** | 0.0 | 0.0 | 0.0 | 0.0 | 0.0 | 0.0 | 0.0 | 0.0 | 0.0 | 0.0 |
| **Retinal Hemorrhage** | 0.0 | 0.0 | 0.0 | 0.0 | 0.0 | 0.0 | 0.0 | 0.0 | 0.0 | 0.0 |
| **Choroidal/Retinal Inflammation** | 0.0 | 0.0 | 0.0 | 0.0 | 0.0 | 0.0 | 0.0 | 0.0 | 0.0 | 0.0 |
| **Fluorescein** | 0.0 | 0.0 | 0.0 | 0.0 | 0.0 | 0.0 | 0.0 | 0.0 | 0.0 | 0.0 |
| **IOP** | 18.0 | 19.3 | 18.0 | 18.0 | 16.0 | 16.0 | 16.0 | 15.3 | 14.3 | 16.0 |
| **Ocular Discomfort – Description** | 0.0 | 0.0 | 0.0 | 0.0 | 0.0 | 0.0 | 0.0 | 0.0 | 0.0 | 0.0 |
| **Ocular Discomfort - Duration** | 0.0 | 0.0 | 0.0 | 0.0 | 0.0 | 0.0 | 0.0 | 0.0 | 0.0 | 0.0 |

| **0.2 mg dose (n=3)** | **Day 0** | | **Day 1** | | **Day 7** | | **Day 14** | | **Day 21** | |
| --- | --- | --- | --- | --- | --- | --- | --- | --- | --- | --- |
| **Ocular observation variables** | **SOCS1** | **CTRL** | **SOCS1** | **CTRL** | **SOCS1** | **CTRL** | **SOCS1** | **CTRL** | **SOCS1** | **CTRL** |
| **Conjunctival Discharge** | 0.3 | 0.3 | 0.3 | 0.3 | 0.3 | 0.3 | 0.3 | 0.3 | 0.3 | 0.3 |
| **Conjunctival Congestion/Hyperemia** | 0.0 | 0.0 | 0.0 | 0.0 | 0.3 | 0.0 | 0.0 | 0.0 | 0.0 | 0.0 |
| **Conjunctival Swelling** | 0.0 | 0.0 | 0.0 | 0.0 | 0.0 | 0.0 | 0.0 | 0.0 | 0.0 | 0.0 |
| **Cornea** | 0.0 | 0.0 | 0.0 | 0.0 | 0.3 | 0.0 | 0.0 | 0.0 | 0.0 | 0.0 |
| **Surface Area of Cornea Involvement** | 0.0 | 0.0 | 0.0 | 0.0 | 0.3 | 0.0 | 0.0 | 0.0 | 0.0 | 0.0 |
| **Vascularization of Cornea** | 0.0 | 0.0 | 0.0 | 0.0 | 0.0 | 0.0 | 0.0 | 0.0 | 0.0 | 0.0 |
| **Menace Response** | + | + | + | + | + | + | + | + | + | + |
| **Pupillary Response** | +/+ | +/+ | +/+ | +/+ | +/+ | +/+ | +/+ | +/+ | +/+ | +/+ |
| **Vertical Pupil Diameter** |  |  |  |  |  |  |  |  |  |  |
| **Aqueous Flare** | 0.0 | 0.0 | 0.0 | 0.0 | 0.0 | 0.0 | 0.0 | 0.0 | 0.0 | 0.0 |
| **Cellular Flare** | 0.0 | 0.0 | 0.0 | 0.0 | 0.0 | 0.0 | 0.0 | 0.0 | 0.0 | 0.0 |
| **Hyphema/Hypopyon** | 0.0 | 0.0 | 0.0 | 0.0 | 0.0 | 0.0 | 0.0 | 0.0 | 0.0 | 0.0 |
| **Iris Involvement** | 0.0 | 0.0 | 0.0 | 0.0 | 0.0 | 0.0 | 0.0 | 0.0 | 0.0 | 0.0 |
| **Lens – Cataract Location** | 0.0 | 0.0 | 0.0 | 0.0 | 0.0 | 0.0 | 0.0 | 0.0 | 0.0 | 0.0 |
| **Lens – Cataract Stage** | 0.0 | 0.0 | 0.0 | 0.0 | 0.0 | 0.0 | 0.0 | 0.0 | 0.0 | 0.0 |
| **Vitreous** | 0.0 | 0.0 | 0.0 | 0.0 | 0.0 | 0.0 | 0.0 | 0.0 | 0.0 | 0.0 |
| **Vitreal Hemorrhage** | 0.0 | 0.0 | 0.0 | 0.0 | 0.0 | 0.0 | 0.0 | 0.0 | 0.0 | 0.0 |
| **Retinal Detachment – Type** | 0.0 | 0.0 | 0.0 | 0.0 | 0.0 | 0.0 | 0.0 | 0.0 | 0.0 | 0.0 |
| **Retinal Detachment – Area** | 0.0 | 0.0 | 0.0 | 0.0 | 0.0 | 0.0 | 0.0 | 0.0 | 0.0 | 0.0 |
| **Retinal Hemorrhage** | 0.0 | 0.0 | 0.0 | 0.0 | 0.0 | 0.0 | 0.0 | 0.0 | 0.0 | 0.0 |
| **Choroidal/Retinal Inflammation** | 0.0 | 0.0 | 0.0 | 0.0 | 0.0 | 0.0 | 0.0 | 0.0 | 0.0 | 0.0 |
| **Fluorescein** | 0.0 | 0.0 | 0.0 | 0.0 | 0.3 | 0.0 | 0.0 | 0.0 | 0.0 | 0.0 |
| **IOP** | 17.0 | 17.3 | 18.3 | 19.0 | 17.3 | 19.7 | 16.7 | 18.0 | 13.7 | 14.3 |
| **Ocular Discomfort – Description** | 0.0 | 0.0 | 0.0 | 0.0 | 0.7 | 0.0 | 0.0 | 0.0 | 0.0 | 0.0 |
| **Ocular Discomfort - Duration** | 0.0 | 0.0 | 0.0 | 0.0 | 1.3 | 0.0 | 0.0 | 0.0 | 0.0 | 0.0 |

 Supplementary Table 6. Physical examination data from vehicle treated (CTRL) and SOCS1-KIR treated (SOCS1) equine eyes over 21 days.

| **1 mg dose (n=3)** | **Day 0** | | **Day 1** | | **Day 7** | | **Day 14** | | **Day 21** | |
| --- | --- | --- | --- | --- | --- | --- | --- | --- | --- | --- |
| **Ocular Observation Variables** | **SOCS1** | **CTRL** | **SOCS1** | **CTRL** | **SOCS1** | **CTRL** | **SOCS1** | **CTRL** | **SOCS1** | **CTRL** |
| **a- wave amplitude (µV)** | -12.1 | -4.7 | -12.0 | -11.1 | -6.0 | -6.6 | -10.1 | -6.4 | -9.7 | -9.1 |
| **a-wave implicit time (ms)** | 13.4 | 13.9 | 12.4 | 13.4 | 13.0 | 12.2 | 12.4 | 13.1 | 13.1 | 13.6 |
| **b-wave amplitude (µV)** | 92.9 | 61.0 | 90.9 | 58.4 | 76.5 | 80.9 | 99.8 | 73.2 | 81.8 | 81.9 |
| **b-wave implicit time (ms)** | 28.7 | 30.1 | 27.9 | 34.1 | 26.7 | 27.2 | 27.0 | 28.0 | 27.0 | 30.1 |

| **2 mg dose (n=3)** | **Day 0** | | **Day 1** | | **Day 7** | | **Day 14** | | **Day 21** | |
| --- | --- | --- | --- | --- | --- | --- | --- | --- | --- | --- |
| **Ocular Observation Variables** | **SOCS1** | **CTRL** | **SOCS1** | **CTRL** | **SOCS1** | **CTRL** | **SOCS1** | **CTRL** | **SOCS1** | **CTRL** |
| **a- wave amplitude (µV)** | -10.3 | -1.2 | -10.3 | -8.0 | -11.0 | -5.9 | -9.3 | -8.5 | -11.0 | -7.3 |
| **a-wave implicit time (ms)** | 12.8 | 11.6 | 12.6 | 13.1 | 12.3 | 12.3 | 12.8 | 13.1 | 11.8 | 13.6 |
| **b-wave amplitude (µV)** | 89.8 | 108.0 | 99.0 | 95.1 | 96.8 | 106.0 | 85.1 | 91.7 | 104.7 | 91.0 |
| **b-wave implicit time (ms)** | 28.4 | 30.0 | 27.5 | 28.4 | 28.0 | 31.3 | 28.1 | 29.3 | 27.6 | 32.1 |

| **0.2 mg dose (n=3)** | **Day 0** | | **Day 1** | | **Day 7** | | **Day 14** | | **Day 21** | |
| --- | --- | --- | --- | --- | --- | --- | --- | --- | --- | --- |
| **Ocular Observation Variables** | **SOCS1** | **CTRL** | **SOCS1** | **CTRL** | **SOCS1** | **CTRL** | **SOCS1** | **CTRL** | **SOCS1** | **CTRL** |
| **a- wave amplitude (µV)** | -12.1 | -9.0 | -8.9 | -7.1 | -9.1 | -9.9 | -11.1 | -7.5 | -7.6 | -6.4 |
| **a-wave implicit time (ms)** | 12.5 | 12.9 | 12.1 | 13.0 | 12.9 | 12.5 | 12.3 | 13.3 | 12.6 | 13.1 |
| **b-wave amplitude (µV)** | 124.7 | 86.6 | 98.4 | 76.2 | 107.5 | 87.7 | 110.5 | 82.2 | 90.7 | 73.7 |
| **b-wave implicit time (ms)** | 27.3 | 28.0 | 27.6 | 29.2 | 28.4 | 28.3 | 27.9 | 32.3 | 29.3 | 31.1 |

Supplementary Table 7. Alpha and beta wave responses in vehicle (CTRL) and SOCS1-KIR (SOCS1) treated equine eyes over 21 days.
